# Controlling Ligand Excimer Formation with Dipole Changes in Emissive Rare-Earth/Phosphonic Acid Complexes

**DOI:** 10.1021/acsomega.5c08830

**Published:** 2025-09-25

**Authors:** Justin C. Johnson, Ross E. Larsen, Iskander Douair, Anastasia Kuvayskaya, Alan Sellinger, Andrew Ferguson

**Affiliations:** 1 Materials, Chemical, and Computational Science Directorate, 53405 National Renewable Energy Laboratory, 15013 Denver West Parkway, Golden, Colorado 80401, United States; 2 Department of Chemistry, 3557 Colorado School of Mines, 1012 14th Street, Golden, Colorado 80401, United States; 3 Renewable and Sustainable Energy Institute, University of Colorado Boulder, Boulder, Colorado 80309, United States

## Abstract

The interactions between substituted arylvinyl phosphonic acid (AVPA) ligands within a Eu-AVPA complex are shown to influence the outcomes of excited state evolution after photoexcitation. Compared with unfunctionalized AVPAs, pairs of ligands functionalized with CF_3_ in the para position preassociate in the ground state of complexes with Eu^3+^ according to calculated geometry optimizations. The CF_3_-substituted AVPA complexes show evidence of red-shifted optical absorption and undergo more efficient excimer formation, as revealed by transient absorption spectroscopy. We rationalize this behavior through simulations of excited-state geometry optimizations that reveal evolution toward interligand phenyl–phenyl planarity for specific excited states. Emission from complexed Eu^3+^ after energy transfer from the ligand is found to be weaker with CF_3_ substitution, which we hypothesize is due to intracomplex, interligand aggregates with excimer-promoting geometries. These observations point to the need to consider ground-state geometries as well as dynamic excited-state processes to understand the flow of energy in rare earth coordination complexes.

## Graphical abstract

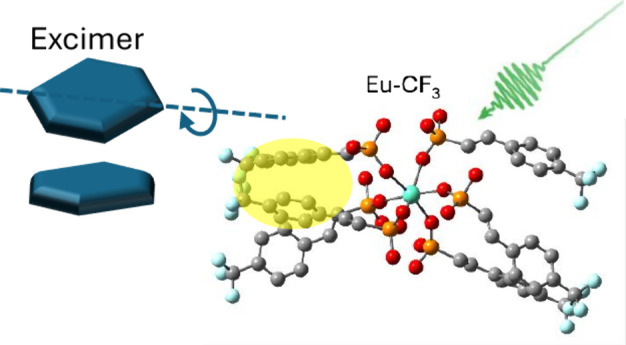

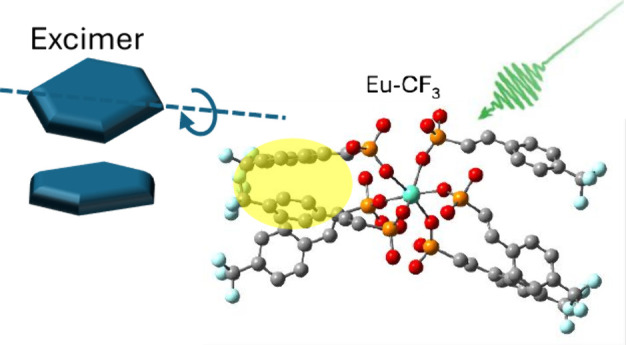

## Introduction

The flow of energy or charge in transition metal complexes from ligand to metal, or vice versa, can be exquisitely controlled through energetic and structural engineering of the components.^1^ Ligands can act as antennae for absorbing light, which is especially important for metal centers with weak d–d or f–f electronic absorption.^2^ The fate of the ligand-centered excited state is then dictated by electronic coupling with the metal center, through either dipole- or exchange-type mechanisms, to induce energy or charge transfer. Bright, metal-centered visible or NIR emission, particularly relevant for rare-earth (RE) elements, or long-lived charge separated states, relevant to photocatalytic metals, are often the goals. With many such photosensitization processes, the interligand interactions are purposefully minimized in order to remove potentially deleterious pathways. However, given sufficient conformational flexibility or lability, organic ligands will often engage in noncovalent intermolecular coupling that is too strong to be ignored.^3,4^ This is particularly true for substituted acenes, whose aromatic π systems are subject to electrostatic influence that results in net attraction.^5–7^ The control of these intermolecular forces specific to a metal–ligand complex geometry is relatively unexplored but may find utility in several applications, including interligand photochemistry toward selective products.^8^

Molecular aggregates are well-studied species in a variety of contexts.^9^ Their formation in the ground state often leads to modification of excited-state properties compared to the behavior of isolated molecules. Symmetry-breaking charge transfer, excimer formation, and exciton coupling are commonly observed phenomena in homogeneous ligand solutions undergoing aggregation.^10,11^ A similar set of properties is likely to arise in metal–ligand complexes, where close proximity is enforced not by the net concentration of ligands but rather by their binding motifs to satisfy the metal coordination sphere. Assuming some degree of flexibility and lability, interligand interactions may lead to ground state interactions within a transition metal complex, and if so, ligand–metal energy or electron flow could be disrupted. Further, if the interligand coupling depends on the identity of the metal (i.e., RE), an opportunity for photodriven separations may become available.^12^

To develop a baseline for controllable interligand behavior around RE complexes, here we investigate and modulate ligand coupling within aryl-vinyl phosphonic acid (AVPA) complexes with Eu.^13^ Compared with a plethora of studies on β-diketonate RE-ligand complexes,^14,15^ phosphonic acids are relatively less explored, particularly with regard to optical properties.^16^ The AVPA ligands (which we refer to by the optically active ‘styryl’ unit in spectroscopic studies) are substituted with −H or −CF_3_ groups, which provide both steric and electrostatic variations. Our prior work investigated the dipole-driven separation of REs using liquid–liquid extraction techniques in the dark with a larger set of substituted styryl ligands.^13^ In that study, the most efficient ligand for RE extraction was the CF_3_ substituted AVPA, and in this paper, we perform spectroscopic studies and density-functional-theory (DFT) and time-dependent DFT (TDDFT) studies of this derivative and the unsubstituted AVPA ligand. We find that the CF_3_ substitution engenders both dipolar and halogen–phenyl electrostatic interactions that drive stacking into excimer-like face-to-face geometries that are evident in transient absorption spectroscopy (TAS) and are seen with DFT geometry optimizations in both the ground and excited electronic states. Different excimer formation pathways lead to unique photophysical outcomes, in terms of energy transfer to Eu^3+^ and its subsequent emission.

## Results and Discussion

### Absorption and Emission

The ligand structures and measured solution-phase absorption spectra for the complexes studied here are shown in Figure 1. The compounds dissolved in chloroform exhibit characteristic UV absorption of styryl derivatives,^17,18^ labeled styryl (unsubstituted) and CF_3_-styryl. The primary absorption band around 250–270 nm is accompanied by weaker side bands from 280 to 300 nm. The peak absorption of CF_3_-styryl is slightly blue-shifted from the styryl ligand, and the side bands are featureless; this shift is also seen in calculated absorption spectra (Figure S1). The optimized geometries suggest that in fact, the CF_3_-styryl would have the largest deviation from planarity (*vide infra*).

**Figure 1:**
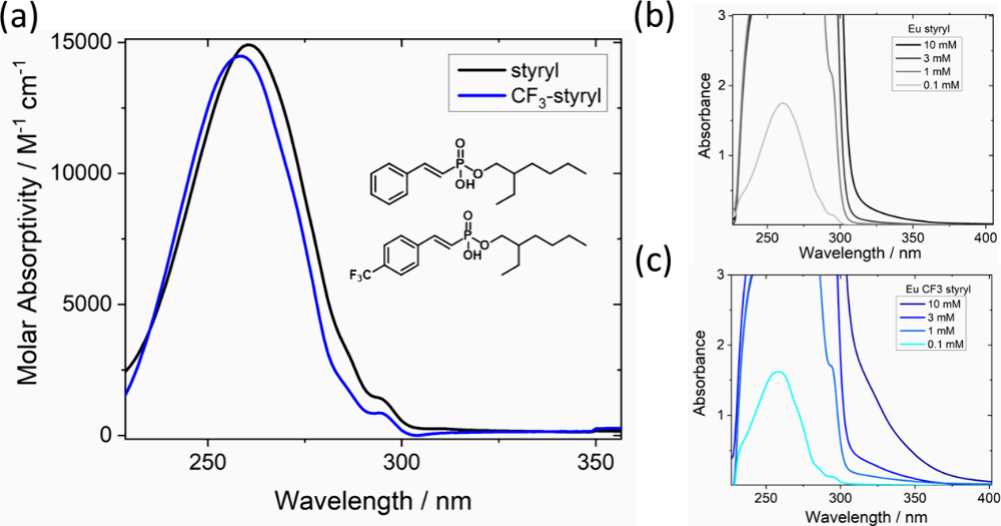
(a) Phosphonic acid ligand structures and absorption in CHCl_3_. UV–vis absorption of (b) Eu-styryl and (c) Eu-CF_3_-styryl complexes vs ligand concentration of ligand in a 1 cm path length quartz cuvette. Ligand is approximately in 8:1 excess, and concentration is based on absorption strength and molar absorptivity of ligand-only solutions.

We note that in RE-ligand solutions, an excess of ligand of roughly 8:1 is present to maintain solubility, which makes complex-specific absorption properties difficult to extract. However, deviations in the line shape at the low energy side of the ligand absorption are evident in highly concentrated Eu-ligand solutions (Figure 1). A broad shoulder beyond 300 nm is observed and is particularly strong for the Eu-CF_3_-styryl complex. The features are also apparent for Eu-styryl but are much weaker than those for Eu-CF_3_-styryl. The ligand-only solutions at similar concentrations do not show a similar shoulder (Figure S2). The absorption at 320 nm is linear in concentration, which disfavors the interpretation of aggregation as the source (Figure S3). Thus, we assign the bands beyond 300 nm as an empirical gauge of ligand–ligand coupling within the Eu complexes, which allows us to specifically excite Eu-ligand complexes separate from the ensemble of free ligands that only absorb <300 nm. It also suggests that the CF_3_-styryl ligands have a stronger propensity to undergo ligand–ligand interactions than does styryl. This observation is corroborated by the weak yellow color of 10 mM Eu-CF_3_-styryl solutions compared with clear solutions of Eu-styryl at similar concentrations. We note that similar red-shifted bands in Eu complexes have been assigned to ligand-to-metal charge-transfer (LMCT) states.^19^ However, in those cases strong π-f interactions are present in the chemical structure, unlike in phosphonic acids that tend to be electronically isolating.^20^

Photoluminescence (PL) spectra collected at two different excitation wavelengths reveal a varying ratio of ligand-related (broad, <550 nm bands) to Eu-related emission (sharp, various bands from 550 to 700 nm), Figure 2a,b. While the former is likely a mixture of fluorescence and phosphorescence (*vide infra*), the latter reports primarily on the efficiency of ligand-Eu energy transfer as the absorption at 320 nm is dominated by ligand transitions. Energy transfer probably occurs from the triplet state of the ligand,^21–24^ which is thermodynamically favored due to the high T_1_ energy of styrene compared with the ^5^D_0_ level of Eu^3+^ (>21 000 cm^–1^ vs 17 250 cm^–1^).^25^ Dual emission of this type has been well-established in related Eu complexes.^26^

**Figure 2:**
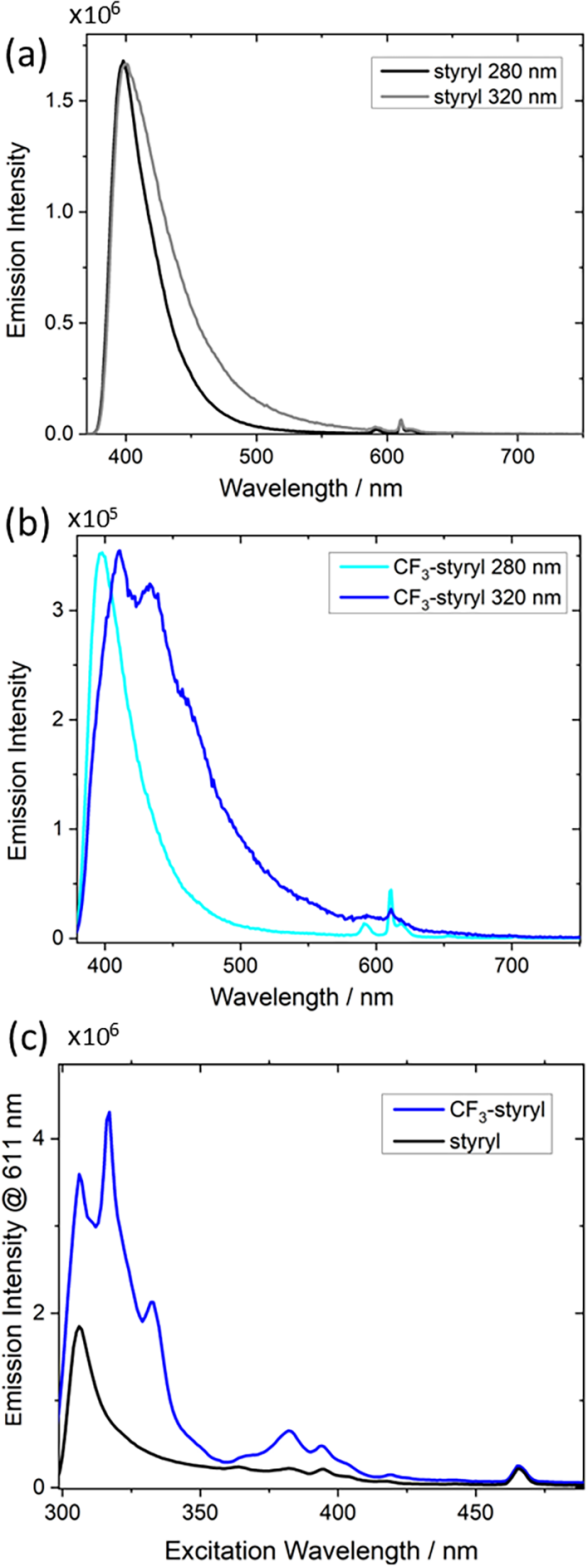
(a, b) Steady-state photoluminescence of Eu complexes in chloroform with two excitation wavelengths. Spectra are normalized at the peak intensity. A 380 nm long pass filter is used to reduce the influence of scattering. (c) Excitation spectrum of complexes, with emission collection at 611 nm.

Variations in the Eu content may alter its emission in the samples of different complexes, but the relative strengths of ligand-centered vs RE emission upon excitation at different wavelengths can be instructive about the energy transfer pathways between the components. Changing from excitation near the ligand peak absorption (280 nm) vs the tail (320 nm) has little effect on the relative intensity of Eu^3+^ vs ligand emission in Eu-styryl complexes, Figure 2a. Some additional broad emission is detected at wavelengths from 450 to 550 nm, likely due to selective excitation of complexed ligands at 320 nm that have a stronger propensity for phosphorescence than styryl AVPAs not proximal to a heavy metal. However, the Eu-CF_3_-styryl complexes exhibit significantly different emission profiles with 280 nm vs 320 nm excitation. The Eu^3+^-derived emission is almost entirely lost upon 320 nm excitation, while the majority of ligand-centered emission shifts to 450–550 nm. Excitation of complexes at the lower energy absorption may target aggregates prone to excimer formation, which typically leads to broad and strongly red-shifted emission.^27,28^ These low-energy species may be less likely to undergo energy transfer to the RE, thus reducing RE emission while also enhancing the broad and red-shifted emission. The photoluminescence excitation (PLE) spectra, Figure 2c, further underscore the distinct behavior in the complexes. When detecting at the strongest Eu emission wavelength (611 nm), the Eu-styryl complex primarily shows a featureless rise below 400 nm, similar to the high-concentration absorption spectrum, Figure 1b. In contrast, the Eu-CF_3_-styryl complex PLE is dominated by sharp transitions associated with direct Eu^3+^ excitation (with some possible contribution from ligands in the underlying broad feature), suggesting that the primary pathway for Eu^3+^ emission is not via energy transfer from the ligand.

### Transient Absorption Spectroscopy

Solutions of each ligand and Eu-ligand complex were investigated with transient absorption (TA) spectroscopy, as shown in Figure 3. Two excitation conditions were employed: 280 nm pump (into the ensemble of mostly free ligands) vs 320 nm pump (primarily Eu-ligand complexes). At 280 nm excitation for the Eu-styryl and Eu-CF_3_-styryl complexes (Figure 3a,b), the primary feature in the TA spectrum is an excited state absorption (ESA) band that peaks below 350 nm (the limit of the spectral sensitivity of the instrument). The feature decays on a 10–100 ps time scale and is virtually identical for both complexes. It is also nearly identical to the behavior of ligand-only solutions excited at 280 nm (Figure S4), and thus we assign this band to the S_1_–S_*n*_ ESA of photoexcited styrene.

**Figure 3:**
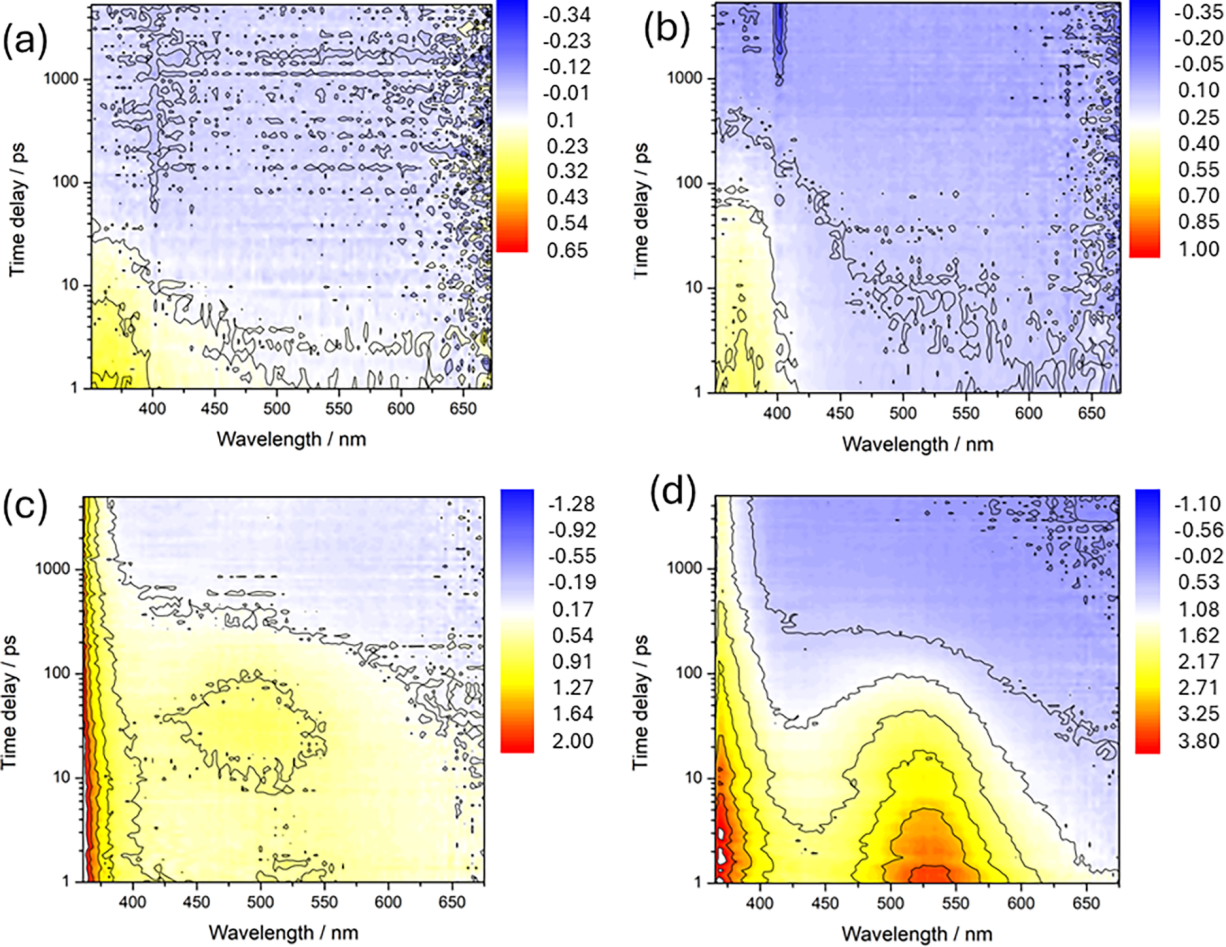
Transient absorption maps for chloroform solutions of (a) Eu-styryl and (b) Eu-CF_3_-styryl at 280 nm excitation. TA maps for 320 nm excitation for (c) Eu-styryl and (d) Eu-CF_3_-styryl. Color legend indicates the ΔmOD.

The results for 320 nm excitation in chloroform are shown in Figure 3c,d for the same two complexes. At this excitation wavelength, the TA spectrum includes the UV ESA and other visible-range ESA bands. Most notably, a band near 500–550 nm emerges, either on a subpicosecond or several picosecond time scale. In the latter case, the band is weak and centered at around 500 nm. Prior studies of (poly)­styrene and related derivatives strongly suggest that the band near 500–550 nm is due to an excimer,^17,18^ while the residual UV ESA bands could be a combination of S_1_–S_*n*_ and T_1_–T_*n*_ transitions.^29^ The latter are likely present due to enhanced intersystem crossing from the proximal Eu via the heavy atom effect. The dominance of the excimer band at the very earliest times for the Eu-CF_3_-styryl signifies the ligand predisposition toward excimer-forming geometries compared with the Eu-styryl complex, which undergoes slower and weak excimer formation. No signal is observed for 320 nm excitation of ligand-only solutions. As a result, we assign any dynamics upon 320 nm excitation as specific to the Eu complex, as we inferred above from the steady-state absorption spectra. We note that the styryl cation also absorbs in this spectral region,^30^ and we cannot rule out the possibility of a CT-excimer that results from partial symmetry breaking charge transfer between ligands. This CT contribution would further stabilize the excimer, leading to a deeper effective trap of the ligand electronic energy. The relatively short lifetime we observe for the excimer (Figure S4c,d) may be another indication of its significant CT character, where recombination to the ground state could be fast.^31^ Annihilation between excimers or between a CT excimer and an exciton may also hasten the return to ground state.^32^ The nonexponential decay of the excimer feature may suggest involvement of bimolecular processes (Figure S4e), although the relatively fast decay of S_1_ in ligand-only solutions points toward intrinsic nonradiative decay routes.

### Ground-State Geometries

DFT calculations were performed to judge interligand geometries in the complexes. We start with the established quasi-octahedral geometry determined from prior studies that confirm six-coordinate Eu as the dominant species for aryl vinyl phosphonic acids ligands.^33–36^ As geometric measures, we computed the interligand distances between the centers of mass (COM) of the rings, between the nearest two atoms between rings (nearest neighbors), and the angle between the planes of the two rings. Computed values for these separations and angles between the aryl rings of each ligand are given in Tables 1 and 2, with details about how the centers of mass and inter-ring angles are defined presented in Supporting Information.

**Table 1:** Selected Inter-Ring Geometric Measures for the Optimized Ground State of the Eu-styryl Complex

Styryl			
ring pair	COM separation (Å)	nearest-neighbor separation (Å)	inter-ring angle (deg)
1–2	7.78	5.59	47
1–3	9.98	8.54	64
2–3	5.29	3.63	58
4–5	4.98	3.82	81
4–6	6.91	5.26	19
5–6	5.07	3.85	85

**Table 2:** Selected Inter-Ring Geometric Measures for the Optimized Ground State of the Eu-CF_3_ Complex

CF_3_-styryl			
ring pair	COM separation (Å)	nearest-neighbor separation (Å)	inter-ring angle (deg)
1–2	4.72	3.49	27
1–3	5.41	3.93	85
2–3	5.41	3.71	64
4–5	5.53	4.06	67
4–6	7.93	7.08	33
5–6	6.35	4.08	40

The ground state geometries reveal that the six ligands attached to the Eu atom divide themselves into two groups of three ligands on opposite sides of the Eu (to the left and right of the central atom in Figure 4), meaning that in terms of interligand interactions we should expect the largest interactions to be within these 3-ligand subgroups. The computed geometries also show the smallest phenyl–phenyl distances between CF_3_-substituted ligands, with an interplanar center-of-mass separation of ∼4.7Å, Figure 4, and a nearest-neighbor separation (closest atoms from the two rings) of ∼3.5 Å and with a small (∼27°) angle between the planes of the closely coordinated rings. In contrast to the CF_3_-subsituted case, twisting or tilting of the styryl ligands leads to either significantly nonparallel phenyls or large center-to-center separations (>4.9 Å) in these complexes, which reduces the interligand electronic coupling. The predicted close styryl–styryl coupling for Eu-CF_3_-styryl, and its absence for other ligands, implicates these geometries as poised to undergo excimer formation upon photoexcitation, as is observed in TA. Further evidence for this hypothesis is seen in the calculated absorption spectra for complexes with styryl and CF_3_-styryl ligands (Figures S7 and S8). In the CF_3_-styryl complex, a relatively bright state is observed ∼9 nm red-shifted from the main absorption peak, which is not seen in the styryl complex, where the most red-shifted bright peak is only ∼6 nm shifted. This slightly red-shifted absorption peak could serve as a spectroscopic indication that the two ligands are interacting in state 57, potentially primed to form an excimer. We tentatively relate this observation to the red-shifted absorption found in Eu-CF_3_-styryl solutions, although the scale of the red shift is not reproduced by calculations. The delayed formation of other excited states in Eu-styryl may be the result of different excimer geometries,^37^ including some that involve motion on a ps time scale (the blue-shifted and delayed-onset bands) that are not fully captured by calculations. An alternative explanation for the slower and weaker formation of excimers in the styryl case is energy transfer among ligands to preferred excimer-forming sites, which may exist at lower density compared with CF_3_-styryl complexes.

**Figure 4:**
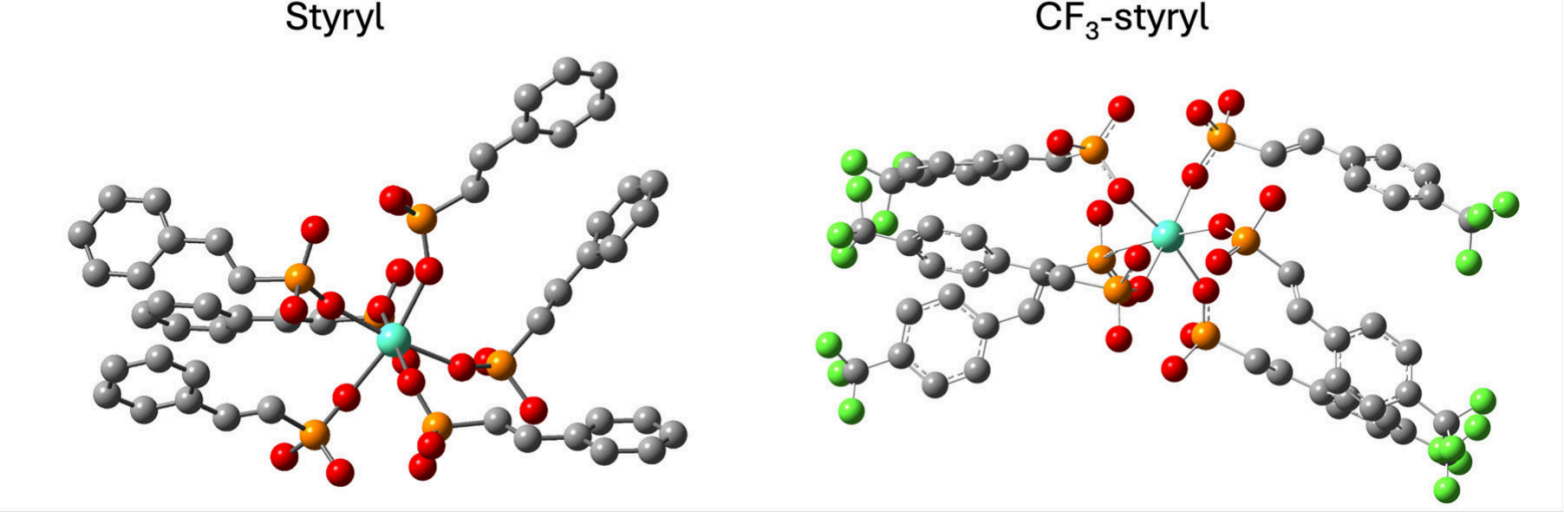
DFT-optimized ground state geometries for Eu-styryl and Eu-CF_3_-styryl complexes. Hydrogens and ethylhexyl solubilizing groups were removed for clarity. Atom types are indicated by sphere color: cyan, europium; red, oxygen; orange, phosphorus; gray, carbon; green, fluorine.

### Excited-State Geometries

To better understand whether the expected larger coupling between CF_3_-substituted aryls does set these complexes up to form excimers, we performed excited-state geometry relaxation calculations on each of the complexes, as described in the computational methods section. Excited-state relaxation calculations required us to calculate the lowest ∼100 excitations for a complex in order to converge excited states up to at least state ∼90. As shown in the Supporting Information (Figures S7 and S8), the calculated absorption spectra for complexes with styryl or substituted ligands are made up of many transitions. We chose the brightest transitions, those with an oscillator strength greater than 0.3 (Tables S5 and S6), and ran calculations to minimize the energy in the excited state. For these calculations, the excited state of a complex was fixed to its initial value (e.g., excited state 57) during the optimization. No attempt was made to track mixing of states from one geometry to the next, so these geometry optimizations should be thought of as optimization on a fixed adiabatic energy surface. These calculations can inform about how the geometry of the complex changes as the ligands move in response to the changed electronic state, but there is no direct comparison to the measured dynamics because a step from a minimization algorithm does not correspond to some approximate measure of time, so these calculations indicate what geometric motions lower the energy of the excited complex, but not how fast the process will be. As discussed above, later evolution toward excited-state geometries (e.g., slow excimer formation) may not be captured by these calculations.

We found that for all the excited states, most of the geometric measures for both styryl and substituted styryl ligands fluctuated during the excited-state geometry optimizations, but the values did not show any secular trend that might indicate the ability to form an excimer. The lone exception was for the CF_3_-styryl complex relaxing in the lowest energy state (state 57) having appreciable oscillator strength. It is noteworthy that CF_3_-styryl state 57 is a lower energy transition with high oscillator strength than is observed in any other ligand, which may hint at the presence of a low energy excitation associated with the two well-aligned rings. We find for state 57 that the angle between rings 1 and 2 starts at ∼27° and after about 10 optimization steps it steadily decreases to less than about 20°, as shown in the top panel of Figure 5. The calculations show that the inter-ring separation between rings 1 and 2 does not shift from its initial ∼4.7 Å value. Table 2 shows that rings 1 and 2 are the two rings that had the smallest separation and best initial alignment among all ligands in the styryl and substituted-styryl complexes. Our calculations indicate that excitation to the 57th excited state causes them to align better as the excited complex relaxes. This result is consistent with the idea that the CF_3_-styryl complex has a ground state geometry that is poised to form an excimer. This geometrical predisposition for subsequent excimer formation, often indicated by a red-shifted absorption from the ground state, is common in solid-state systems with potential for strong face-to-face interactions or dipole-driven electrostatic interactions.^38^ We speculate that the initial association is dipole-driven^13^ and that additional electrostatic forces in the excited-state, such as π–π and CH–F interactions, may further promote the conditions for dominant excimer formation. Interestingly, in solid-state samples of Eu^3+^ compounds with aromatic ligands, similar interactions may also drive intermolecular structure,^20^ although for the compounds studied here crystallization was not achieved.

**Figure 5:**
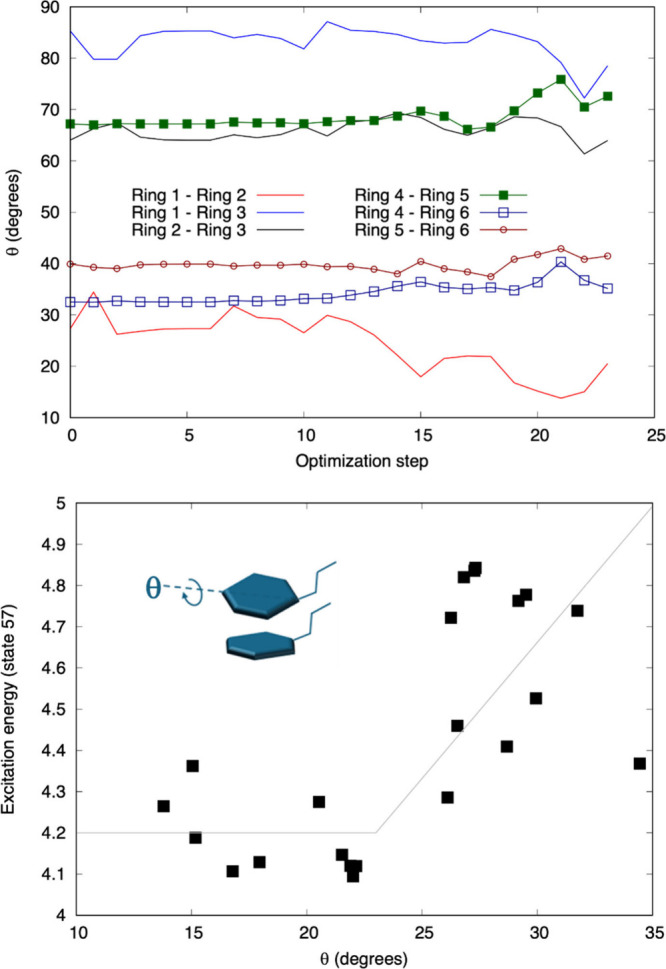
Top panel: Inter-ring angles as a function of excited-state optimization step for excited state 57 of the CF_3_-styryl ligand complex. Bottom panel: Correlation of excited state 57 energy with angle between rings one and two for geometries taken from each optimization step; the gray line serves to guide the eye.

Further evidence that alignment of the rings can be associated with an increased inter-ring coupling can be found by correlating the excitation energy from the ground state to state 57 with the inter-ring angle for the geometries of each step in the optimization. The lower panel of Figure 5 shows that below about 23° the excitation energy is reduced and becomes somewhat constant as a function of the angle. This is consistent with the idea that two of the rings in the 6-ligand shell of the CF_3_-styryl complex are electronically coupled enough to support a state shared between two ligands when the inter-ring angle becomes less than about 23°. The large scatter in excitation energy likely arises because the excitation energy of a ligand, or of a coupled ligand pair, depends on many coordinates, bond lengths, bond angles, etc. and not solely on one inter-ring angle.

Figure 6 summarizes the overall photophysical picture based on experimental and computational results. The styryl and CF_3_-styryl complexes are shown to undergo separate pathways based on their differing abilities to sustain interligand states, as demonstrated in Figure 3. These states at least partially reduce the efficiency of transfer to the emissive Eu center through either energetic or spatial localization mechanisms. This efficiency reduction is reflected in the different emission spectra in Figure 2 for the two complexes.

**Figure 6:**
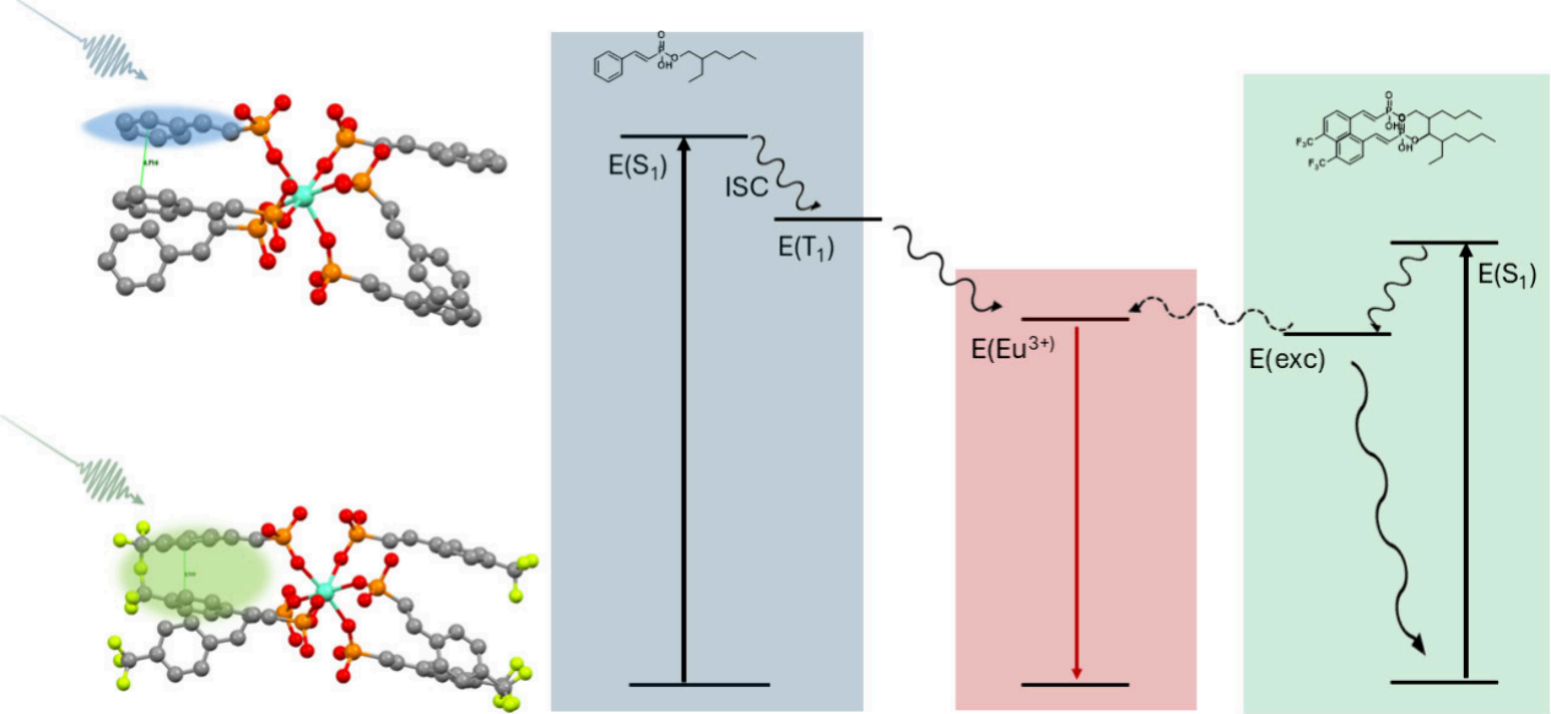
Styryl (upper, blue) and CF_3_-styryl (lower, green) excitation and excited-state evolution pathways, showing reduced likelihood of Eu emission (red) upon shifted excitation of ligand aggregates.

## Conclusion

We have shown that excimer formation is strongly dependent on the substitution of the styryl ligand in Eu^3+^-AVPA complexes. Preassociated ligands in complexes are evident as red-shifted absorption in steady-state experiments (allowing specific photoselection of complexes from the mixture of free ligands) and as a new, slightly red-shifted peak in TDDFT calculations. Proximal vinylphenyl groups in DFT calculations of Eu-CF_3_-styryl, the ligand with a large dipole moment, are implicated as the primary source of the intracomplex ligand aggregate behavior. These proximal groups are found, in calculations of the excited state relaxation, to align and show improved inter-ring electronic coupling during relaxation on the state associated with the slightly red-shifted peak. The resulting excimers are observed in the TA data most strongly for Eu-CF_3_-styryl, and the excimer pathway likely reduces the efficiency of energy transfer to the emissive Eu center, as judged by steady-state PL experiments. Control of the bifurcated excited state pathway through further ligand design and its complexation with different RE elements may provide a unique route to photodriven separation of critical materials.

## Experimental Section

### Extraction Procedure

Aqueous Eu­(NO_3_)_3_ stock solution was diluted to 0.01 M, and the pH was adjusted to 2.0 with HNO_3_. 0.1 M ligand solution was prepared using CHCl_3_ as a solvent. 1.5 mL of each solution was added to the centrifuge tube, vortexed for 2 min, and centrifuged to separate phases. Styryl phosphonates resulted in formation of distinct clear phases, while CF_3_ ligands produced a small amount of precipitate at the bottom of the tube and cloudy organic phase.

### Spectroscopy

Chloroform used for solutions for spectroscopic experiments was used as-received from Sigma-Aldrich. Absorption spectra were collected on a Cary 7000 spectrophotometer. Photoluminescence spectra were obtained with a Horiba Fluoromax spectrometer.

Transient absorption data sets were acquired using a Coherent Libra Ti:sapphire laser, with an output of 800 nm at 1 kHz. A TOPAS-C OPA was used to generate the ∼150 fs pump pulse tuned from 280 to 340 nm for these studies to excite the peak and shoulder of the sample absorption. The pump pulse energy was typically ∼100 nJ, and the pump spot size was found to be approximately 300 μm obtained by using a beam profiler. In an Ultrafast Systems Helios Spectrometer, a small amount of 800 nm light was used to pump a 1 mm thick CaF_2_ crystal to generate 350–800 nm probe light for UV–vis TA. A delay of up to 5 ns can be achieved with the Helios. Delay times earlier than 1 ps contain a coherent artifact due to pump–probe interactions in the solvent, thus the time window is truncated.

### Calculations

Density functional theory calculations were performed to compute both the ground state (DFT) and excited state (TDDFT) properties of the three Eu-aryl complexes discussed in this paper. All calculations were done with the Gaussian 16 electronic structure program, revision C.01 ^39^ using the range-corrected ωB97X exchange correlation functional.^40,41^ The short-range ω value was modified from its standard value of 0.3 bohr^–1^ to 0.2 bohr^–1^ following benchmark calculations, in which we found that the standard value gave values for the spin density that were too large for Eu and Nd complexes with aryl-containing ligands. Reducing the value of ω to 0.2 bohr^–1^ gave the correct spin values across a range of lanthanide-aryl complexes. We used the 6-31G­(d, p) double-z Pople-type basis set for the H, C, O, and F atoms.^42^ The P and Eu atoms were represented with the small-core Stuttgart–Dresden relativistic effective core potentials associated with their adapted basis set.^43–45^ To model the P atom’s valence orbitals, its basis was augmented with a d-polarization function (α = 0.387).^46^ The ground-state geometry of each complex was optimized using default Gaussian settings, and subsequently a single-point time-dependent DFT (TDDFT) calculation of the first 100 excited states was performed. The computed excitation energies and transition dipoles were used to calculate the absorption spectra. Subsequently, for each complex we ran geometry optimization with TDDFT for all excited states with oscillator strengths greater than 0.3, as listed in Tables S3 and S4 in the Supporting Information.

Geometry optimizations in the chosen excited electronic states were computationally intensive, and we were unable to fully converge the geometries in any of the excited states. Hence, the optimization runs show trends, but they do not arrive at the final gas-phase excited state geometries. We emphasize, however, that the electronic structure was fully converged but that the large number of degrees of freedom, most of which are not likely to be consequential, showed continued evolution at the termination of the optimization runs. To understand the initial steps in relaxation following photoexcitation that would reflect how the ground-state geometry might enhance excimer formation, we typically ran between 20 and 25 optimization steps for each complex on each chosen excited state, with some optimization runs achieving more steps and two runs (states 76 and 77 for the CF_3_-styryl system) taking only 13 and 12 steps, respectively. Excited state energies as a function of minimization step for all of the selected excited states are shown in Figures S9–S26 of the Supporting Information. As noted above, the default Gaussian convergence criteria for forces and atom-move distances were not achieved for any of the excited state optimization runs. The default convergence criteria used by Gaussian and the level of convergence achieved for each run are reported in the Supporting Information.

## Supplementary Material



## Notes

Absorption spectra and transient data for ligands, calculated ground-state geometrical coordinates for complexes, and calculated spectra and excited-state relaxation (PDF)
