# Supplementary material for: Controlling Ligand Excimer Formation with Dipole Changes in Emissive Rare-Earth/Phosphonic Acid Complexes
Source: ACS Omega. 2025 Sep 25;10(39):46188–96. doi: 10.1021/acsomega.5c08830 (PMC12508948; doi:10.1021/acsomega.5c08830)
Supplement: Supplementary file 1 [file ao5c08830_si_001.pdf]

**Supplementary Information for:**

# Controlling ligand excimer formation with dipole changes in emissive rare-earth/phosphonic acid complexes

Justin C. Johnson[1,3,*] Ross E. Larsen,[1,3,*] Iskander Douair,[1] Anastasia Kuvayskaya,[2] Alan Sellinger,[2] Andrew Ferguson[1,3]

1. Materials, Chemical, and Computational Science Directorate, National Renewable Energy Laboratory, 15013 Denver West Pkwy, Golden, CO 80401
2. Department of Chemistry, Colorado School of Mines, 1012 14th St., Golden, CO 80401
3. Renewable and Sustainable Energy Institute, University of Colorado Boulder, Boulder, Colorado 80309

## I. Calculated absorption spectra

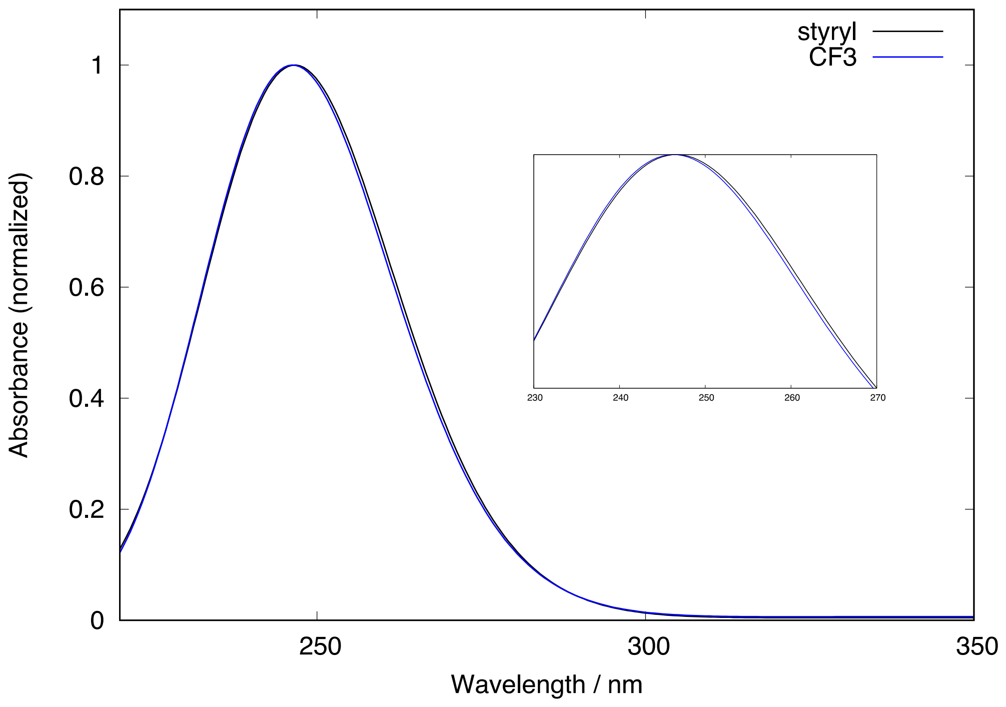


**Figure S1**: Computed absorption spectra normalized to the peak height for complexes with styryl and $CF_3$-styryl ligands from optimized ground state geometries, using TDDFT, no

solvation (gas phase). The computed spectrum is broadened in energy by a gaussian of width 0.25 eV. The inset shows an expanded scale near the absorption peaks.

## II. Additional Spectroscopic Data

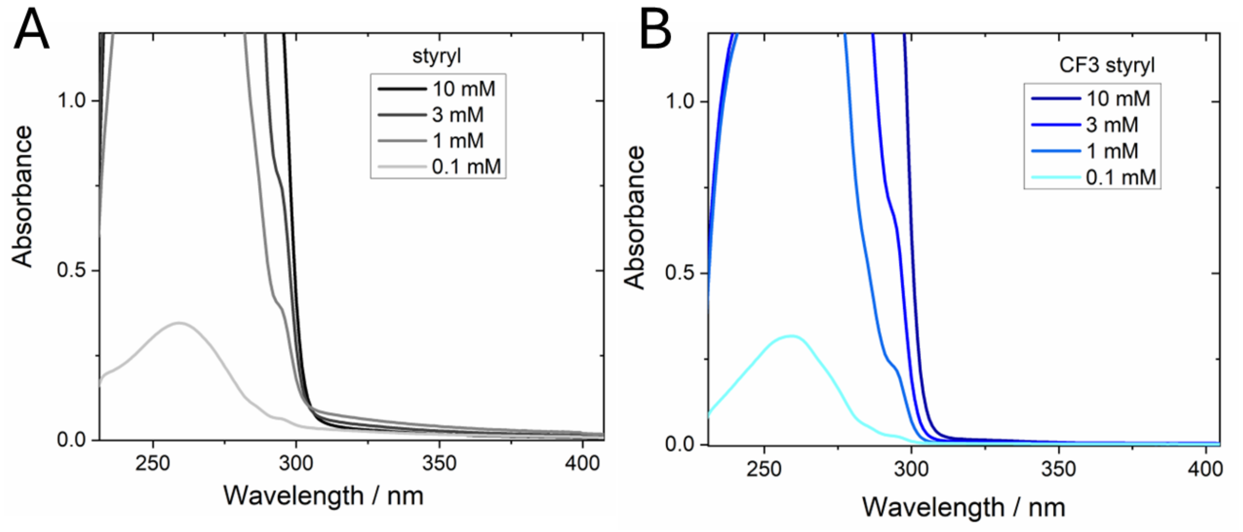


**Figure S2**. Concentration dependence of ligand absorption for (A) styryl and (B) $CF_3$-styryl ligand solutions in chloroform. Spectra were obtained in a 2 mm quartz cuvette.

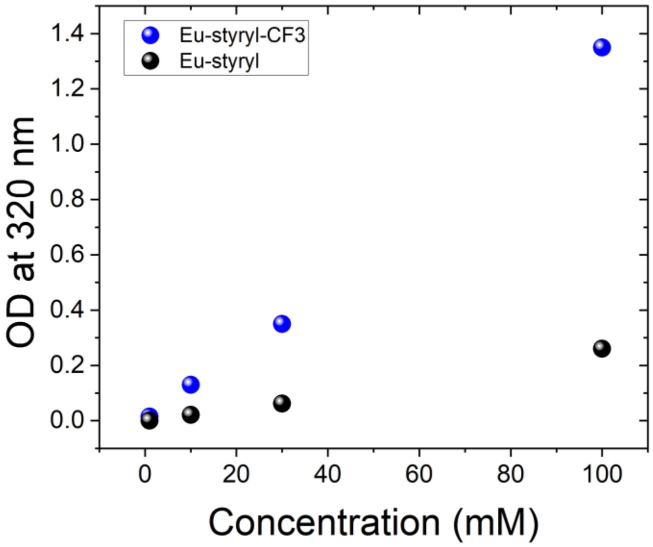


**Figure S3**. Optical absorption at 320 nm for various concentrations of complexes in chloroform.

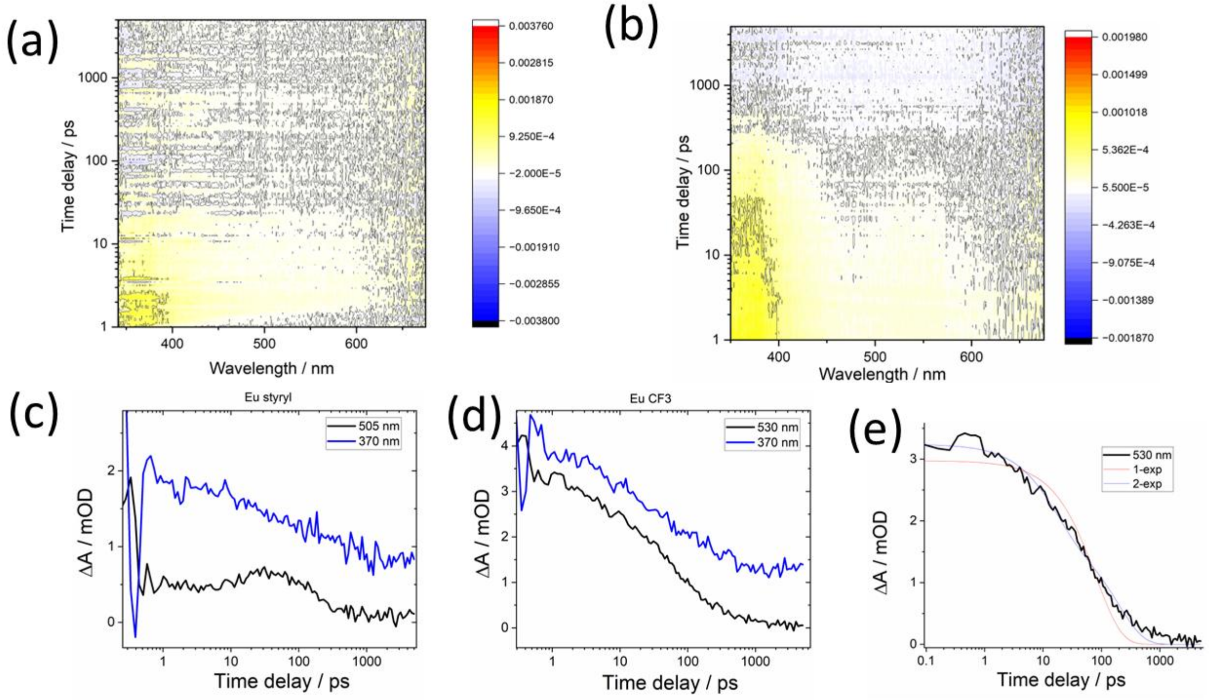


**Figure S4**. Transient absorption data for (a) styryl and (b) $CF_3$-styryl ligand in $CHCl_3$, excited at 280 nm. Color scale is ΔOD. Sliced kinetic traces from TA data for (c) styryl and (d) $CF_3$-styryl, showing excimer and singlet/triplet population kinetics. (e) One and two exponential fits to 530 nm decay kinetics for $CF_3$-styryl.

## III: Computational Results

### Structural images and ground-state optimized coordinates

<u>Styryl</u>

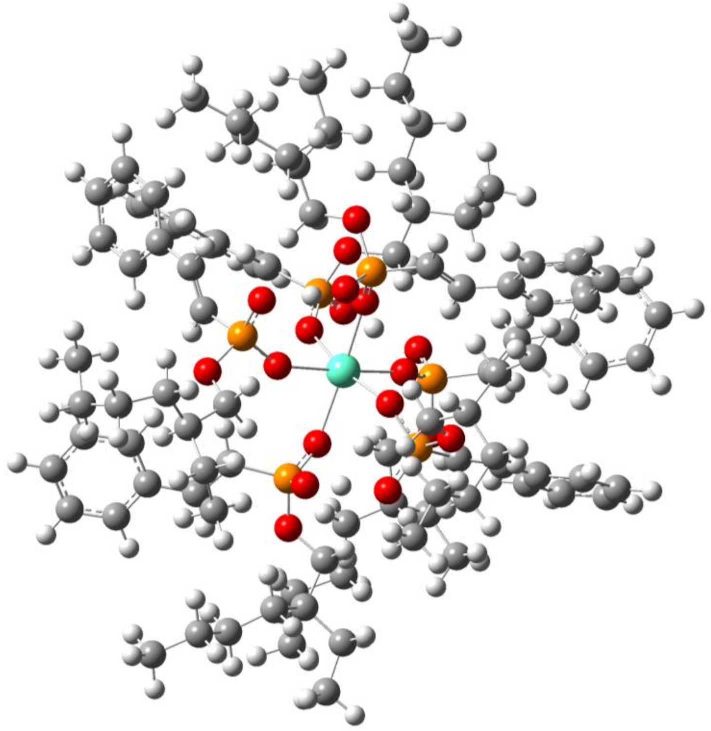

**Figure S5**: Optimized geometry of Eu-styryl complexes. Gray spheres are carbon atoms, white spheres are hydrogen atoms, red spheres are oxygen atoms, orange spheres are phosphorous atoms, and the teal sphere is a europium atom. Bonds and bond types are calculated according to the distance criteria in the Gauss View 6 visualization program.

Table S1. Computed optimized structure for styryl complex (in Å).

| | | | |
|---|---|---|---|
| Eu | 0.05529792 | -0.0629826 | -0.1994543 |
| P | -1.8578991 | -3.0619802 | -0.5427163 |
| P | 1.79387238 | -2.7725375 | 1.47698969 |
| P | 0.89108081 | 2.44122421 | 2.39872186 |
| P | -3.0295002 | 1.00310147 | 1.5594804 |
| P | -0.1137775 | 2.62919584 | -2.7040954 |
| P | 2.96195358 | -0.2229702 | -2.594943 |
| O | 0.61853035 | 4.04806052 | 2.26138133 |
| O | -1.5775636 | -3.5018829 | 0.95868709 |
| O | -1.9106095 | -4.3992811 | -1.4850148 |
| O | -0.8343509 | -2.0383414 | -1.0928862 |
| O | 2.64311728 | -2.7740748 | 2.88533759 |
| O | 1.1401445 | -1.3668426 | 1.30662051 |
| O | 0.78878369 | -3.9514393 | 1.43354596 |
| O | -0.079473 | 1.84595474 | 3.51693461 |

| | | | |
|---|---|---|---|
| O | 0.7683213 | 1.7676543 | 1.02042846 |
| O | -4.2372854 | -0.0201681 | 2.04467731 |
| O | -1.9936362 | 0.12747234 | 0.78654966 |
| O | -2.4902778 | 1.85039236 | 2.72599035 |
| O | -0.6157076 | 1.45092425 | -1.8457332 |
| O | 0.76359786 | 3.69477529 | -1.8166702 |
| O | 0.72085529 | 2.22216126 | -3.9941317 |
| O | 2.83807247 | -1.7685539 | -3.1859412 |
| O | 2.77195798 | 0.82799079 | -3.7044969 |
| O | 1.99387154 | -0.1126449 | -1.3789628 |
| C | 3.34984669 | 9.7035896 | -1.2064796 |
| C | 2.76250275 | 8.41963422 | -1.7983717 |
| C | 3.17905918 | 7.15986345 | -1.0297889 |
| C | 2.6121547 | 5.87652819 | -1.6480307 |
| C | 4.85610527 | 4.80865305 | 0.75346052 |
| C | 4.27953516 | 4.23027757 | -0.5440305 |
| C | 2.79935666 | 4.59215887 | -0.8204753 |
| C | -4.1658448 | 7.65467909 | 5.48901073 |
| C | -2.8915291 | 6.85766133 | 5.19748846 |
| C | -2.5760559 | 6.75824057 | 3.70115021 |
| C | -1.3216827 | 5.92657744 | 3.40246944 |
| C | -0.1593451 | 8.33854475 | 1.77480876 |
| C | 0.16844263 | 6.86768599 | 1.50642354 |
| C | -0.9364012 | 5.87005201 | 1.90364283 |
| C | -4.5891815 | -4.2565278 | 2.89313481 |
| C | -4.8763586 | -3.1942829 | 3.96115993 |
| C | -8.1306412 | 0.57834367 | 2.94750325 |
| C | -8.230206 | -0.8488049 | 3.49772381 |
| C | -7.3055682 | -1.117293 | 4.69768347 |
| C | -5.811642 | -0.8625259 | 4.44158892 |
| C | -5.1525244 | -1.7866477 | 3.39876199 |
| C | 0.72891561 | -6.1251275 | 6.84411567 |
| C | 1.5911195 | -4.865632 | 6.71970685 |
| C | 2.59652274 | -4.9505912 | 5.56403482 |
| C | 3.56119756 | -3.7584186 | 5.46764058 |
| C | 4.88827924 | -1.064211 | 6.28034967 |
| C | 3.95666596 | -1.259621 | 5.0807054 |
| C | 2.90916516 | -2.3774072 | 5.2526242 |
| C | -6.5211921 | -7.9764932 | -0.0071848 |
| C | -5.1271587 | -7.5567917 | 0.46659676 |

| | | | |
|---|---|---|---|
| C | -4.1190832 | -7.4242013 | -0.6793237 |
| C | -2.7379105 | -6.9417118 | -0.2184673 |
| C | -1.2961657 | -9.3412229 | -1.6008971 |
| C | -0.6775954 | -7.955526 | -1.3961821 |
| C | -1.7037206 | -6.8072484 | -1.3632398 |
| C | -1.6672251 | -7.3222985 | -5.5786995 |
| C | -1.4553247 | -5.8379523 | -5.2706816 |
| C | 0.01515701 | -5.4818659 | -5.0212717 |
| C | 3.4263205 | -5.2035984 | -5.1382049 |
| C | 2.71872819 | -3.8483575 | -5.2589826 |
| C | 0.20377814 | -4.0081097 | -4.6443369 |
| C | 1.62178424 | -3.6015705 | -4.1982666 |
| C | -4.6504622 | 6.2580296 | -6.5190288 |
| C | -4.6796496 | 6.10253597 | -5.1293108 |
| C | -3.6385179 | 5.64857699 | -7.2627084 |
| C | -3.7054546 | 5.34459245 | -4.4841331 |
| C | -2.6615895 | 4.89094947 | -6.6164632 |
| C | -2.6796184 | 4.72754024 | -5.2212675 |
| C | 7.65941878 | -4.2531815 | -2.0163832 |
| C | 7.82407218 | -4.243807 | -0.6294374 |
| C | 6.4036703 | -3.9710742 | -2.5654592 |
| C | 6.738451 | -3.9552986 | 0.19976479 |
| C | 5.31847711 | -3.6859218 | -1.7374919 |
| C | 5.47094891 | -3.6733733 | -0.3376599 |
| C | 9.65596361 | 0.84924739 | -1.1150206 |
| C | 8.97243666 | -0.3708041 | -1.0828666 |
| C | 9.00012398 | 1.99920325 | -1.5624666 |
| C | 7.6393819 | -0.4410642 | -1.4869692 |
| C | 7.67036332 | 1.92717248 | -1.9790329 |
| C | 6.96947201 | 0.71059148 | -1.9369887 |
| C | -8.7145732 | -2.0664038 | -1.0714721 |
| C | -8.2803858 | -3.2197573 | -1.7270991 |
| C | -7.7923766 | -1.2663477 | -0.3879054 |
| C | -6.9292456 | -3.5674519 | -1.7006638 |
| C | -6.4424246 | -1.612014 | -0.361016 |
| C | -5.9924475 | -2.7679898 | -1.025735 |
| C | 7.68655546 | 1.48955116 | 3.2289138 |
| C | 6.80411774 | 1.94072424 | 4.21695439 |
| C | 7.19617085 | 1.09770669 | 1.98202118 |
| C | 5.43758689 | 2.0094194 | 3.95621818 |

| | | | |
|---|---|---|---|
| C | 5.82541185 | 1.1569308 | 1.72537657 |
| C | 4.93074584 | 1.62366673 | 2.70245436 |
| C | -6.4072271 | 6.11168011 | -1.758851 |
| C | -5.6997349 | 6.53747549 | -0.6327441 |
| C | -6.3919043 | 4.75927681 | -2.1179156 |
| C | -4.9900283 | 5.61269587 | 0.13396992 |
| C | -5.6773984 | 3.8379803 | -1.3539336 |
| C | -4.9654852 | 4.25130813 | -0.2135074 |
| C | 3.50282747 | 1.72133083 | 2.3552856 |
| C | 2.55078275 | 2.36310737 | 3.05580906 |
| C | -0.5503345 | 4.45300345 | 1.48841761 |
| C | -4.5766745 | -3.1798686 | -1.0322654 |
| C | 5.56058568 | 0.66441577 | -2.3811633 |
| C | 4.36077848 | -3.3774024 | 0.59204246 |
| C | -4.2171027 | 3.31101397 | 0.64496056 |
| C | -1.6135717 | 3.92292715 | -4.5976154 |
| C | -3.9479936 | 2.0224034 | 0.38397016 |
| C | -3.5424664 | -2.4558352 | -0.5762413 |
| C | 3.10613449 | -3.0304098 | 0.26273086 |
| C | 4.65777067 | -0.2479882 | -1.9889347 |
| C | -1.4734804 | 3.63800648 | -3.2927724 |
| C | 2.15581401 | 3.39888721 | -1.5282523 |
| C | 1.57277666 | -2.1309327 | -3.7814643 |
| C | -3.8658734 | -1.1499503 | 2.87269569 |
| C | 1.93165074 | -2.3759185 | 4.0774297 |
| C | -0.9587333 | -5.4750861 | -1.2840816 |
| H | -5.742204 | -0.9906609 | 0.19749265 |
| H | -6.5895579 | -4.4676281 | -2.2126309 |
| H | -5.6901141 | 2.78669975 | -1.6350821 |
| H | -4.4496025 | 5.94492104 | 1.02047907 |
| H | -3.7499884 | 5.2356856 | -3.4016242 |
| H | -1.8706194 | 4.41688092 | -7.1964333 |
| H | 7.16236154 | 2.82285882 | -2.3350432 |
| H | 7.12586141 | -1.4014378 | -1.4823059 |
| H | 4.75980702 | 2.35548504 | 4.73489362 |
| H | 5.44242518 | 0.84900523 | 0.75202593 |
| H | 6.86919956 | -3.9489967 | 1.28135205 |
| H | 4.3548943 | -3.4504443 | -2.1879268 |
| H | 4.447251 | 9.67779352 | -1.2163421 |
| H | 3.03310485 | 9.83844996 | -0.163755 |

| | | | |
|---|---|---|---|
| H | 3.03164573 | 10.589199 | -1.7690252 |
| H | 3.07186027 | 8.32226752 | -2.8499338 |
| H | 1.66402923 | 8.48681212 | -1.8098725 |
| H | 4.27804803 | 7.11104532 | -0.9954132 |
| H | 2.84191362 | 7.24746618 | 0.0160392 |
| H | 3.06450201 | 5.72373362 | -2.6423992 |
| H | 1.53574754 | 6.01277574 | -1.8260125 |
| H | 4.25716585 | 4.48767667 | 1.61517938 |
| H | 4.87068767 | 5.90412759 | 0.7490744 |
| H | 5.88244742 | 4.45647966 | 0.9133805 |
| H | 4.3788273 | 3.13336105 | -0.4953039 |
| H | 4.8922203 | 4.5445402 | -1.4039189 |
| H | 2.28539055 | 4.71582955 | 0.1479177 |
| H | -5.0392082 | 7.19060673 | 5.0126918 |
| H | -4.0865788 | 8.68103556 | 5.10740491 |
| H | -4.3658073 | 7.71344026 | 6.5652605 |
| H | -2.9851748 | 5.84411032 | 5.61439098 |
| H | -2.0386296 | 7.32271641 | 5.71442462 |
| H | -3.4381611 | 6.3038005 | 3.1841476 |
| H | -2.471502 | 7.77112941 | 3.28288427 |
| H | -1.4872947 | 4.90498665 | 3.77535702 |
| H | -0.4728081 | 6.31812414 | 3.9834867 |
| H | -0.2871988 | 8.53870241 | 2.84602793 |
| H | -1.0827301 | 8.64241365 | 1.26381623 |
| H | 0.64806254 | 8.98771412 | 1.41442011 |
| H | 1.09094591 | 6.5925505 | 2.03841836 |
| H | 0.38084734 | 6.7372355 | 0.43386964 |
| H | -1.8344337 | 6.10395228 | 1.30324033 |
| H | -3.6762438 | -4.0408699 | 2.32451613 |
| H | -5.4224854 | -4.3184934 | 2.1792411 |
| H | -4.4655901 | -5.2460139 | 3.3503314 |
| H | -5.7473619 | -3.510159 | 4.55250753 |
| H | -4.0362073 | -3.139121 | 4.67178435 |
| H | -8.9008211 | 0.76392075 | 2.18780396 |
| H | -7.1523187 | 0.76798181 | 2.48734258 |
| H | -8.27138 | 1.32109957 | 3.74443218 |
| H | -8.0203971 | -1.5646548 | 2.68896111 |
| H | -9.2653122 | -1.0468574 | 3.81065531 |
| H | -7.6242629 | -0.475902 | 5.53343375 |
| H | -7.457715 | -2.1520931 | 5.03940744 |

| | | | |
|---|---|---|---|
| H | -5.6728178 | 0.18018522 | 4.12613095 |
| H | -5.2628865 | -0.9671833 | 5.39106423 |
| H | -5.8250762 | -1.8924947 | 2.53175573 |
| H | 1.35040559 | -7.0121637 | 7.02332404 |
| H | 0.15785675 | -6.3023432 | 5.92377961 |
| H | 0.01389581 | -6.0446629 | 7.67145258 |
| H | 2.13520652 | -4.6941553 | 7.6614892 |
| H | 0.93547141 | -3.99251 | 6.5849213 |
| H | 3.19496461 | -5.8660932 | 5.68699012 |
| H | 2.05645373 | -5.074573 | 4.6134519 |
| H | 4.26475439 | -3.9387566 | 4.64184784 |
| H | 4.16435008 | -3.7212436 | 6.386546 |
| H | 5.53273601 | -1.9353985 | 6.44807127 |
| H | 4.31842913 | -0.8880855 | 7.20285866 |
| H | 5.54426334 | -0.2000567 | 6.11911691 |
| H | 4.5506679 | -1.4622943 | 4.17797854 |
| H | 3.42329469 | -0.3170105 | 4.88407652 |
| H | 2.3006745 | -2.1324572 | 6.13990462 |
| H | -6.4886743 | -8.9401592 | -0.5321339 |
| H | -6.940765 | -7.235177 | -0.7001278 |
| H | -7.2181273 | -8.0766038 | 0.83302412 |
| H | -5.1927026 | -6.5969384 | 1.00095111 |
| H | -4.7491108 | -8.2868322 | 1.19799401 |
| H | -4.0278418 | -8.3895729 | -1.2016874 |
| H | -4.5115912 | -6.7158353 | -1.4281461 |
| H | -2.8606757 | -5.9707681 | 0.28278228 |
| H | -2.3474571 | -7.6256903 | 0.55144888 |
| H | -1.8745924 | -9.3827276 | -2.5335958 |
| H | -1.9703394 | -9.6095391 | -0.7780565 |
| H | -0.5190857 | -10.112392 | -1.6545353 |
| H | -0.1007103 | -7.9458558 | -0.4586309 |
| H | 0.04635424 | -7.7649235 | -2.2035374 |
| H | -2.2429441 | -6.8093168 | -2.3262635 |
| H | -1.3249388 | -7.9496745 | -4.7439479 |
| H | -1.1052403 | -7.6271427 | -6.4710303 |
| H | -2.7248699 | -7.5505167 | -5.755819 |
| H | -1.8398709 | -5.2274629 | -6.1013948 |
| H | -2.0463744 | -5.5528901 | -4.3866532 |
| H | 0.40948927 | -6.1239728 | -4.2153756 |
| H | 0.60310632 | -5.7254705 | -5.9190826 |

| | | | |
|---|---|---|---|
| H | 2.73287252 | -6.0469005 | -5.2363141 |
| H | 3.91986262 | -5.2978404 | -4.1612771 |
| H | 4.19599523 | -5.3157336 | -5.9118258 |
| H | 3.47509799 | -3.05751 | -5.1668348 |
| H | 2.28042283 | -3.739915 | -6.2632788 |
| H | -0.092182 | -3.3809668 | -5.5014839 |
| H | -0.4989322 | -3.7632013 | -3.831821 |
| H | 1.87598377 | -4.178436 | -3.2913572 |
| H | -5.413293 | 6.85232211 | -7.0180633 |
| H | -5.4640295 | 6.57492123 | -4.5411641 |
| H | -3.6082178 | 5.76361819 | -8.344145 |
| H | 8.50233405 | -4.4777678 | -2.6667553 |
| H | 8.79568272 | -4.4655747 | -0.1918421 |
| H | 6.26761078 | -3.9691159 | -3.6452798 |
| H | 10.6971947 | 0.90120523 | -0.8027952 |
| H | 9.47768324 | -1.2761227 | -0.75151 |
| H | 9.52659468 | 2.95089312 | -1.594041 |
| H | -9.7677075 | -1.793048 | -1.0865247 |
| H | -8.9921959 | -3.8494739 | -2.2568178 |
| H | -8.1261393 | -0.3707524 | 0.13288657 |
| H | 8.75409692 | 1.44158198 | 3.43457276 |
| H | 7.18366725 | 2.24056833 | 5.19183325 |
| H | 7.87402055 | 0.74530526 | 1.20686585 |
| H | -6.9780415 | 6.82729557 | -2.3480873 |
| H | -5.7080328 | 7.58672765 | -0.3453416 |
| H | -6.9424938 | 4.42114704 | -2.9934269 |
| H | 3.21245872 | 1.2428881 | 1.4167667 |
| H | 2.73906577 | 2.88445423 | 3.99188917 |
| H | -0.2880167 | 4.39377759 | 0.42358772 |
| H | -1.3771771 | 3.76015014 | 1.69498416 |
| H | -4.3725863 | -4.1654513 | -1.4547528 |
| H | 5.24077708 | 1.43791452 | -3.0827443 |
| H | 4.60755686 | -3.4581489 | 1.65172266 |
| H | -3.8556087 | 3.71883702 | 1.59215892 |
| H | -0.8638497 | 3.52659062 | -5.2841841 |
| H | -4.2516291 | 1.53224181 | -0.5407823 |
| H | -3.6457296 | -1.4580181 | -0.1518608 |
| H | 2.78419277 | -2.9141037 | -0.7736884 |
| H | 4.89723288 | -1.0261672 | -1.2643385 |
| H | -2.1738917 | 3.97032977 | -2.527784 |

| | | | |
|---|---|---|---|
| H | 2.20366488 | 2.51020794 | -0.8872454 |
| H | 2.67399049 | 3.18317527 | -2.4740376 |
| H | 1.39126265 | -1.4849368 | -4.6535933 |
| H | 0.76644008 | -1.9781998 | -3.0501978 |
| H | -3.243313 | -0.7980865 | 3.70965076 |
| H | -3.2728418 | -1.8510117 | 2.27111574 |
| H | 1.51194878 | -1.371972 | 3.92564605 |
| H | 1.1022073 | -3.0762461 | 4.24781765 |
| H | -0.4547365 | -5.3625673 | -0.3148137 |
| H | -0.2044847 | -5.3952823 | -2.0779471 |
| H | -0.5424206 | -3.6519839 | 1.19778018 |
| H | -1.0622371 | 1.79334365 | 3.20856925 |
| H | 1.5495011 | 1.60212134 | -3.8427681 |

CF3-styryl

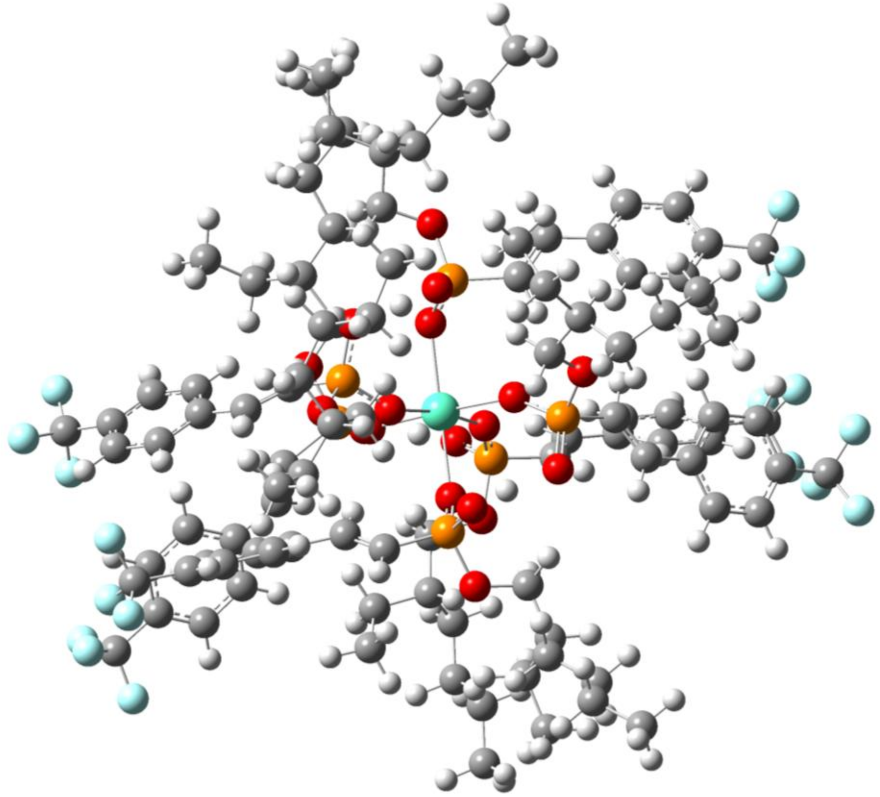

**Figure S6**: Optimized geometry of Eu-$CF_3$-styryl complexes. Gray spheres are carbon atoms, white spheres are hydrogen atoms, red spheres are oxygen atoms, orange spheres are phosphorous atoms, and the teal sphere is a europium atom. Bonds and bond types are calculated according to the distance criteria in the Gauss View 6 visualization program.

Table S2. Computed optimized structure for $CF_3$-styryl complex (in Å).

| | | | |
|---|---|---|---|
| Eu | 0.02138989 | 0.33808227 | -0.0291988 |
| P | -2.4948769 | -0.6304247 | -2.6084246 |
| P | 1.28660403 | -2.4616798 | -2.1131545 |
| P | 2.9715779 | 0.51214917 | 2.2225373 |
| P | -0.6520228 | -1.6427704 | 2.90843425 |
| P | -2.4775811 | 2.58962911 | 1.27925645 |
| P | 1.0279695 | 3.6687503 | -0.7827722 |
| F | -9.4746429 | 1.37165353 | -4.2088474 |
| F | -9.6194695 | -0.5574853 | -3.2167453 |
| F | -10.738894 | 1.14075404 | -2.446983 |
| F | -10.169344 | -5.3843427 | -2.9289814 |
| F | -9.2953607 | -6.5432872 | -1.3088024 |
| F | -10.261651 | -4.6323677 | -0.8918843 |

| | | | |
|---|---|---|---|
| F | -9.4256681 | 0.20184093 | 2.37790895 |
| F | -9.6742496 | -1.8812303 | 1.79492495 |
| F | -9.4605133 | -1.3485085 | 3.89901068 |
| F | 10.7273893 | -3.1699893 | -0.3017881 |
| F | 10.2925043 | -4.8181174 | 1.05861439 |
| F | 9.51986899 | -4.8521133 | -0.9708361 |
| F | 10.1210446 | 3.02174001 | -1.7656005 |
| F | 9.80972847 | 2.47208269 | 0.32224211 |
| F | 9.35729412 | 1.0482247 | -1.2581888 |
| F | 9.77263674 | -3.8759592 | -4.6114058 |
| F | 9.18248924 | -5.9552889 | -4.399164 |
| F | 9.01173438 | -4.9712621 | -6.3353022 |
| O | -1.7922409 | -0.3132992 | -1.2499221 |
| O | -1.723855 | -1.514168 | -3.601541 |
| O | -2.9280063 | 0.81374859 | -3.2950774 |
| O | 0.59153248 | -2.4812065 | -3.5445781 |
| O | 0.66503104 | -3.6631133 | -1.1945917 |
| O | 1.21129977 | -1.0983336 | -1.3976938 |
| O | 2.48154147 | -0.3000301 | 3.4336659 |
| O | 1.90218862 | 0.8673378 | 1.1409234 |
| O | 3.68667559 | 1.9381762 | 2.66342335 |
| O | -0.2709957 | -1.45709 | 1.42721517 |
| O | 0.01275863 | -0.5966224 | 3.91356674 |
| O | -1.900831 | 3.9921013 | 0.95267456 |
| O | -1.406757 | 1.45713337 | 1.33243372 |
| O | -3.2781892 | 2.60871668 | 2.71473827 |
| O | 0.31129177 | 2.3575676 | -1.195127 |
| O | 0.9130553 | 4.81111789 | -1.9420069 |
| O | 0.5030709 | 4.26930913 | 0.58962839 |
| O | -0.2634149 | -3.1524767 | 3.4009298 |
| C | -0.3054367 | 5.59416651 | -2.0536952 |
| C | -2.4504815 | 2.78186757 | 3.89011295 |
| C | 2.86223487 | 2.99353347 | 3.21512709 |
| C | -1.896296 | 1.5348827 | -4.0071587 |
| C | -0.7760853 | -3.700469 | -1.0164259 |
| C | 2.97918254 | -2.9922917 | -2.3567206 |
| C | -4.1249622 | -1.3220459 | -2.2554241 |
| C | -3.785608 | 2.1717912 | 0.11009116 |
| C | 2.78906321 | 3.40902551 | -0.5907335 |
| C | 4.39846614 | -0.2594283 | 1.42660747 |

| | | | |
|---|---|---|---|
| C | 3.48425041 | -3.1807982 | -3.5857672 |
| C | 4.968092 | -1.3670889 | 1.92267005 |
| C | -5.069491 | 1.99524705 | 0.45613944 |
| C | -4.4539741 | -2.5476268 | -2.6924112 |
| C | 3.75911396 | 3.9922006 | -1.3085273 |
| C | 1.12950056 | -3.5521807 | 3.25919368 |
| C | -2.4165322 | -1.5563312 | 3.20384674 |
| C | -3.267193 | -1.4072209 | 2.17577891 |
| C | 6.17895635 | -2.0344778 | 1.40105163 |
| C | 7.05167981 | -1.4146362 | 0.48883568 |
| C | 6.50351091 | -3.3179916 | 1.87184521 |
| C | 8.21411207 | -2.054675 | 0.06626233 |
| C | 7.6610946 | -3.9667881 | 1.44866527 |
| C | 8.52132941 | -3.3311053 | 0.5494358 |
| C | -4.7347244 | -1.314172 | 2.31214118 |
| C | -5.556088 | -1.80482 | 1.28537305 |
| C | -5.3341711 | -0.7501457 | 3.45114275 |
| C | -6.9447956 | -1.7602926 | 1.40180943 |
| C | -6.7198351 | -0.6912701 | 3.56620193 |
| C | -7.525648 | -1.202524 | 2.54225602 |
| C | 5.19015426 | 3.65132462 | -1.1601187 |
| C | 5.68393762 | 3.02812494 | 0.00154352 |
| C | 6.07856584 | 3.91591165 | -2.2132521 |
| C | 7.01732121 | 2.64023455 | 0.0818197 |
| C | 7.41520148 | 3.52301395 | -2.1383505 |
| C | 7.88172146 | 2.87462784 | -0.9937948 |
| C | -5.7450343 | -3.2251133 | -2.4537095 |
| C | -5.7962903 | -4.6282888 | -2.4571661 |
| C | -6.9332116 | -2.5062637 | -2.2335669 |
| C | -6.9917646 | -5.3009177 | -2.210881 |
| C | -8.1333509 | -3.1739434 | -2.0021628 |
| C | -8.1603471 | -4.572496 | -1.9786151 |
| C | -6.1878174 | 1.69872583 | -0.4626478 |
| C | -6.0471441 | 1.68047272 | -1.8632894 |
| C | -7.451633 | 1.43170116 | 0.08774727 |
| C | -7.1404008 | 1.40465532 | -2.6810611 |
| C | -8.5472021 | 1.14700602 | -0.7246914 |
| C | -8.3926887 | 1.14023177 | -2.1128543 |
| C | 4.86721079 | -3.5733041 | -3.9140108 |
| C | 5.17668541 | -3.8727533 | -5.2491422 |

| | | | |
|---|---|---|---|
| C | 5.88704253 | -3.6630731 | -2.9485066 |
| C | 6.46348136 | -4.2646492 | -5.6177403 |
| C | 7.17054752 | -4.0542521 | -3.3090423 |
| C | 7.4586709 | -4.3574182 | -4.6460833 |
| C | -2.5094754 | 2.79436647 | -4.6210367 |
| C | -1.3727877 | 3.60835539 | -5.2692395 |
| C | -3.6605695 | 2.44742473 | -5.5941717 |
| C | -4.8329536 | 3.43569369 | -5.5671474 |
| C | -1.7741826 | 4.98638654 | -5.8084855 |
| C | -0.5691684 | 5.82636444 | -6.2482159 |
| C | -0.9646385 | 7.19639528 | -6.8052816 |
| C | 0.06533695 | 7.05783585 | -2.2883847 |
| C | -1.204723 | 7.82593921 | -2.7026585 |
| C | -0.967077 | 9.29010996 | -3.0815051 |
| C | 0.77324934 | 7.66471563 | -1.0533269 |
| C | 1.98843596 | 8.53829179 | -1.3905432 |
| C | 2.62666745 | 9.18334505 | -0.1558521 |
| C | 3.86234839 | 10.0232177 | -0.4902131 |
| C | -3.2928123 | 3.37864221 | 5.0171687 |
| C | -4.3547553 | 2.37006375 | 5.49697445 |
| C | -5.1575759 | 2.81889557 | 6.72207438 |
| C | -3.9139018 | 4.73544649 | 4.62634155 |
| C | -2.9467564 | 5.77066312 | 4.03013217 |
| C | -1.7831019 | 6.16416054 | 4.94935876 |
| C | -0.9000417 | 7.25585703 | 4.33790749 |
| C | 3.79905034 | 4.09147184 | 3.72087752 |
| C | 4.58787461 | 3.5986137 | 4.9499569 |
| C | 5.88648041 | 4.36794956 | 5.24259474 |
| C | 6.97612697 | 4.25048336 | 4.16247166 |
| C | 7.41597159 | 2.81087748 | 3.87398627 |
| C | 3.01792251 | 5.38886025 | 4.00855302 |
| C | 2.62670745 | 6.17772337 | 2.75371704 |
| C | 1.36842967 | -4.7901113 | 4.12081083 |
| C | 0.57011057 | -5.9868227 | 3.5687422 |
| C | 0.80471413 | -7.3152867 | 4.29184962 |
| C | 1.09882664 | -4.4936518 | 5.6162816 |
| C | 2.13946664 | -5.089007 | 6.57426053 |
| C | 1.82108696 | -4.8199214 | 8.04905766 |
| C | 2.87631257 | -5.3882857 | 9.00175096 |
| C | -1.1548319 | -4.8899088 | -0.1368346 |

| | | | |
|---|---|---|---|
| C | -2.688879 | -4.8572349 | 0.08091821 |
| C | -3.1288572 | -5.2056167 | 1.50719228 |
| C | -0.6472576 | -6.2078826 | -0.7482956 |
| C | -0.9521444 | -7.4711244 | 0.06463523 |
| C | -0.3218428 | -8.7322219 | -0.5381586 |
| C | -0.6239001 | -9.9959567 | 0.27143936 |
| C | -9.5592439 | 0.78387438 | -2.9966082 |
| C | -9.4731005 | -5.2860679 | -1.7735745 |
| C | -9.0226808 | -1.0713267 | 2.65250067 |
| C | 9.77178478 | -4.0364217 | 0.08865334 |
| C | 9.29738563 | 2.36469797 | -0.9232622 |
| C | 8.85841365 | -4.7909916 | -5.0049146 |
| H | -0.3419022 | -2.0294807 | -3.5598623 |
| H | 1.01276511 | -0.4253187 | 3.71828182 |
| H | -0.5496259 | 4.11297804 | 0.77919884 |
| H | -0.8767897 | 5.18979211 | -2.8990992 |
| H | -0.9087057 | 5.48583483 | -1.1417298 |
| H | -1.6098046 | 3.44619074 | 3.64837959 |
| H | -2.0398785 | 1.80298973 | 4.17395711 |
| H | 2.18137582 | 3.36011317 | 2.43487512 |
| H | 2.26088986 | 2.58888843 | 4.04353606 |
| H | -1.0936591 | 1.80752661 | -3.306262 |
| H | -1.4819159 | 0.87971847 | -4.7868375 |
| H | -1.2497127 | -3.7802685 | -2.006775 |
| H | -1.1012619 | -2.7671731 | -0.5410924 |
| H | 3.56350321 | -3.107943 | -1.4455504 |
| H | -4.8021236 | -0.714072 | -1.6542522 |
| H | -3.4382703 | 2.07849757 | -0.9203231 |
| H | 2.99712464 | 2.63463474 | 0.14638323 |
| H | 4.77599419 | 0.24482731 | 0.53649152 |
| H | 2.8166564 | -3.045574 | -4.4380331 |
| H | 4.51564958 | -1.8193082 | 2.8075369 |
| H | -5.3391408 | 2.077511 | 1.50998734 |
| H | -3.709685 | -3.1043777 | -3.2656749 |
| H | 3.49984275 | 4.72358014 | -2.0751181 |
| H | 1.77775473 | -2.7246673 | 3.58053634 |
| H | 1.31916901 | -3.7568961 | 2.19627563 |
| H | -2.7482114 | -1.6352146 | 4.23757964 |
| H | -2.872165 | -1.3631978 | 1.15746764 |
| H | 8.89623539 | -1.5475551 | -0.6103976 |

| | | | |
|---|---|---|---|
| H | 7.90842906 | -4.9535027 | 1.83166237 |
| H | -7.5703234 | -2.1570118 | 0.60650797 |
| H | -7.176152 | -0.2496053 | 4.44911324 |
| H | 7.38949774 | 2.16657999 | 0.98787869 |
| H | 8.09227075 | 3.71910839 | -2.9653407 |
| H | -7.0187842 | -6.3871259 | -2.1980913 |
| H | -9.0480483 | -2.605943 | -1.8538786 |
| H | -7.0235986 | 1.40386634 | -3.7622308 |
| H | -9.5155404 | 0.93884516 | -0.277536 |
| H | 6.69301213 | -4.4962342 | -6.6537557 |
| H | 7.94931004 | -4.1311908 | -2.5545426 |
| H | -2.9232371 | 3.38503849 | -3.7849492 |
| H | -0.5658706 | 3.73925258 | -4.5296526 |
| H | -0.9337175 | 3.01648391 | -6.088776 |
| H | -3.2586742 | 2.36942729 | -6.6160628 |
| H | -4.0456315 | 1.45146248 | -5.339285 |
| H | -5.6177411 | 3.13483314 | -6.2717656 |
| H | -5.2828759 | 3.47846056 | -4.5658034 |
| H | -4.5257852 | 4.45496524 | -5.8289056 |
| H | -2.4621333 | 4.87213821 | -6.6595821 |
| H | -2.3361231 | 5.53601428 | -5.0345837 |
| H | 0.10999315 | 5.95768639 | -5.3908854 |
| H | 0.003502 | 5.27007132 | -7.0052801 |
| H | -0.0849261 | 7.77599078 | -7.1084954 |
| H | -1.6159076 | 7.0937379 | -7.6827201 |
| H | -1.5107377 | 7.78594234 | -6.05644 |
| H | 0.76621809 | 7.07140264 | -3.1405748 |
| H | -1.6685168 | 7.30910472 | -3.5572218 |
| H | -1.9349795 | 7.77491352 | -1.880158 |
| H | -1.9002078 | 9.76292989 | -3.409124 |
| H | -0.5836171 | 9.87174855 | -2.2343434 |
| H | -0.242984 | 9.37395588 | -3.9026638 |
| H | 0.04578319 | 8.25255616 | -0.4710226 |
| H | 1.10464434 | 6.85652208 | -0.3855342 |
| H | 2.73895749 | 7.91831724 | -1.9069329 |
| H | 1.70428474 | 9.32555258 | -2.1057218 |
| H | 1.87872715 | 9.81239978 | 0.34992865 |
| H | 2.89714859 | 8.39591216 | 0.56385042 |
| H | 4.29671355 | 10.4749904 | 0.40917591 |
| H | 4.63993387 | 9.41058609 | -0.9646452 |

| | | | |
|---|---|---|---|
| H | 3.61221237 | 10.8357518 | -1.1846911 |
| H | -2.5885758 | 3.53966858 | 5.85129564 |
| H | -3.8426024 | 1.42397848 | 5.73502288 |
| H | -5.0383759 | 2.15371673 | 4.66198855 |
| H | -5.8145525 | 2.01535639 | 7.07581947 |
| H | -4.492548 | 3.09310526 | 7.55164574 |
| H | -5.7907263 | 3.6857829 | 6.50121959 |
| H | -4.3899941 | 5.1693894 | 5.51767211 |
| H | -4.7198548 | 4.5488032 | 3.90196795 |
| H | -2.5533318 | 5.40855284 | 3.06859697 |
| H | -3.5247436 | 6.67639676 | 3.79195069 |
| H | -1.1610671 | 5.28321363 | 5.17040282 |
| H | -2.1801831 | 6.50500631 | 5.91817883 |
| H | -0.0646124 | 7.51747236 | 4.99890303 |
| H | -0.4805936 | 6.92568183 | 3.37844914 |
| H | -1.4771433 | 8.17049304 | 4.14943967 |
| H | 4.50642199 | 4.29645695 | 2.90028962 |
| H | 3.92928508 | 3.65810641 | 5.83084186 |
| H | 4.82165012 | 2.53392773 | 4.81785846 |
| H | 5.66271661 | 5.43285917 | 5.40615459 |
| H | 6.29725867 | 3.99864656 | 6.19427702 |
| H | 7.84696801 | 4.83665098 | 4.4877919 |
| H | 6.6352895 | 4.71965326 | 3.2276249 |
| H | 7.72690677 | 2.29926829 | 4.79465832 |
| H | 6.60297328 | 2.22158751 | 3.42868948 |
| H | 8.26773548 | 2.79213892 | 3.18230867 |
| H | 2.11785887 | 5.14561484 | 4.59616711 |
| H | 3.63138147 | 6.03060549 | 4.65628617 |
| H | 2.09946202 | 7.10181825 | 3.02097617 |
| H | 3.52216032 | 6.4584067 | 2.18211427 |
| H | 1.97059092 | 5.60330009 | 2.08794834 |
| H | 2.44288796 | -5.0158284 | 4.00117399 |
| H | 0.82813673 | -6.1080423 | 2.50499395 |
| H | -0.5002186 | -5.7355302 | 3.59812661 |
| H | 0.25052317 | -8.1252525 | 3.80208967 |
| H | 1.86759262 | -7.5909289 | 4.29030061 |
| H | 0.46965495 | -7.2750948 | 5.3355814 |
| H | 0.09568496 | -4.8579366 | 5.88516934 |
| H | 1.06666697 | -3.4052636 | 5.77319745 |
| H | 2.22670673 | -6.1743329 | 6.41448638 |

| | | | |
|---|---|---|---|
| H | 3.12919163 | -4.6675925 | 6.33517132 |
| H | 0.83689957 | -5.2491999 | 8.28994512 |
| H | 1.7269534 | -3.7350487 | 8.20644536 |
| H | 2.62373567 | -5.1846215 | 10.0488219 |
| H | 2.96867184 | -6.4759481 | 8.88589538 |
| H | 3.86306 | -4.9495565 | 8.80511898 |
| H | -0.6652034 | -4.7372062 | 0.83926673 |
| H | -3.173247 | -5.5353832 | -0.6386216 |
| H | -3.0703896 | -3.8516082 | -0.1610817 |
| H | -4.2194423 | -5.147879 | 1.60879065 |
| H | -2.8176456 | -6.2175048 | 1.79287964 |
| H | -2.6849649 | -4.506612 | 2.22793044 |
| H | 0.43918798 | -6.1269641 | -0.8931493 |
| H | -1.0815035 | -6.3152947 | -1.7567727 |
| H | -0.5881334 | -7.3432904 | 1.09722339 |
| H | -2.0404988 | -7.6188329 | 0.14097507 |
| H | 0.76687122 | -8.5919549 | -0.6119538 |
| H | -0.6824187 | -8.8593983 | -1.5699873 |
| H | -0.161705 | -10.882255 | -0.1785216 |
| H | -0.2445865 | -9.9067076 | 1.29796028 |
| H | -1.7046567 | -10.177609 | 0.33325244 |
| H | -5.0791856 | 1.87207989 | -2.3247043 |
| H | -7.5791361 | 1.43981849 | 1.16816745 |
| H | -5.1053674 | -2.237956 | 0.39275638 |
| H | -4.7095811 | -0.3276133 | 4.23536559 |
| H | -6.926606 | -1.4180516 | -2.2748439 |
| H | -4.8862776 | -5.1960213 | -2.6457102 |
| H | 4.39810424 | -3.8015982 | -6.0069107 |
| H | 5.68488539 | -3.416503 | -1.9077212 |
| H | 5.84457814 | -3.8056657 | 2.58857118 |
| H | 6.84685909 | -0.4075536 | 0.13019138 |
| H | 5.71426547 | 4.41432537 | -3.1101052 |
| H | 5.02943227 | 2.84846005 | 0.85410689 |

**Geometric inter-aryl properties of optimized ground state structures**

The six ligands attached to the Eu atom, cluster into two sets of three roughly on opposite sides of the central Eu atom. We number the ligands (and the ring in each ligand) as 1-3 for one set of neighbors and 4-6 for the other set. Below we tabulate several measures of the aryl ring geometries within the sets. We do not show geometric measures for rings between the different sets because the rings are separated by large distances, so this is not considered of interest.

In Tables 1 and 2 of the main text, the geometric measures for inter-ligand properties are defined as:

- COM separation refers to the distance between the centers of mass of each ring, with the center of mass computed including hydrogen atoms.
- Nearest-neighbor separation refers to the smallest distance among all inter-ring pairs of atoms (including hydrogens).
- Inter-ring angle is found by taking the dot product between two unit vectors that are normal to the plane of each ring. The unit vectors are found from the normalized cross product of two vectors that point along two (non-parallel) carbon-carbon bonds in each ring and then the inverse cosine is taken to get the angle.

**Spectra and transition strengths**

The optical absorption spectra found from TDDFT calculations for the ground-state optimized geometries of each structure are shown below. Black curves are the spectra broadened with a gaussian broadening in energy of the 0.333 eV for the half-width at half-height. The blue spikes indicate the oscillator strength at the transition wavelengths for all 100 computed excited states.

Tables S7-S9 list all excited states with oscillator strength greater than 0.3. These excited states were chosen for excited-state geometry optimization discussed in the paper.

<u>Styryl</u>

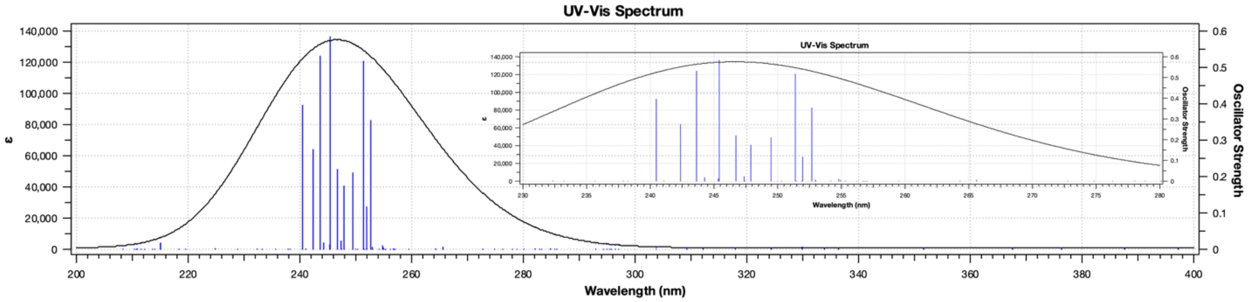


**Figure S7**: TDDFT computed excitation spectrum and oscillator strengths for the lowest 100 transitions. Inset shows a smaller range of wavelengths near the computed peak.

| Styryl | | |
|---|---|---|
| Excited state number | Excitation Wavelength (nm) | Oscillator Strength |
| 62 | 253 | 0.35 |
| 66 | 251 | 0.52 |
| 74 | 245 | 0.58 |
| 77 | 244 | 0.53 |
| 80 | 240 | 0.40 |

*Table S5: Excited states for ground-state geometry styryl complex with oscillator strengths greater than 0.3.*

CF$_3$-styryl

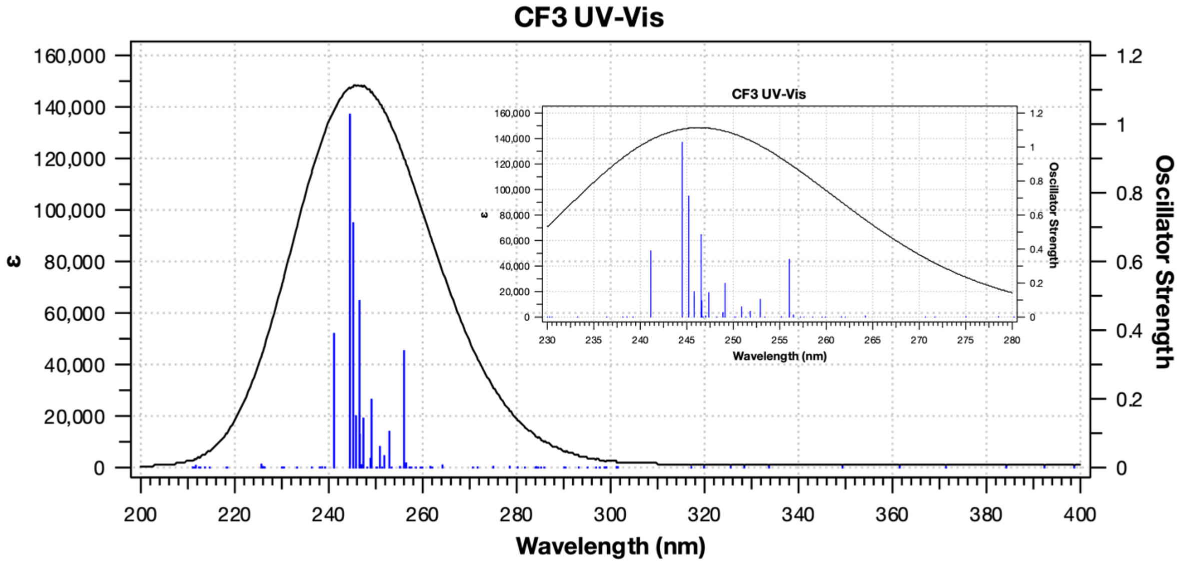


**Figure S8**: TDDFT computed excitation spectrum and oscillator strengths for the lowest 100 transitions. Inset shows a smaller range of wavelengths near the computed peak.

| CF$_3$-Styryl | | |
|---|---|---|
| Excited state number | Excitation Wavelength (nm) | Oscillator Strength |
| 57 | 256 | 0.34 |
| 73 | 247 | 0.48 |
| 75 | 245 (244.5) | 0.71 |
| 76 | 245 (245.2) | 1.0 |
| 77 | 241 | 0.39 |

*Table S6: Excited states for ground-state geometry CF$_3$-styryl complex with oscillator strengths greater than 0.3.*

**Energy relaxation in excited states**

Values of the geometric convergence criteria that are found at the final step of each excited state optimization run are given in the figure captions below. The standard convergence thresholds for geometry optimizations in Gaussian are:

| Criterion | Threshold |
|---|---|
| Maximum Force | 0.000450 |
| RMS Force | 0.000300 |
| Maximum Displacement | 0.001800 |
| RMS Displacement | 0.001200 |

Note that geometries in the excited-state geometry optimization runs are not fully converged, but the energies for each geometry during the optimization run are converged at each step.

<u>Styryl</u>

State 62:

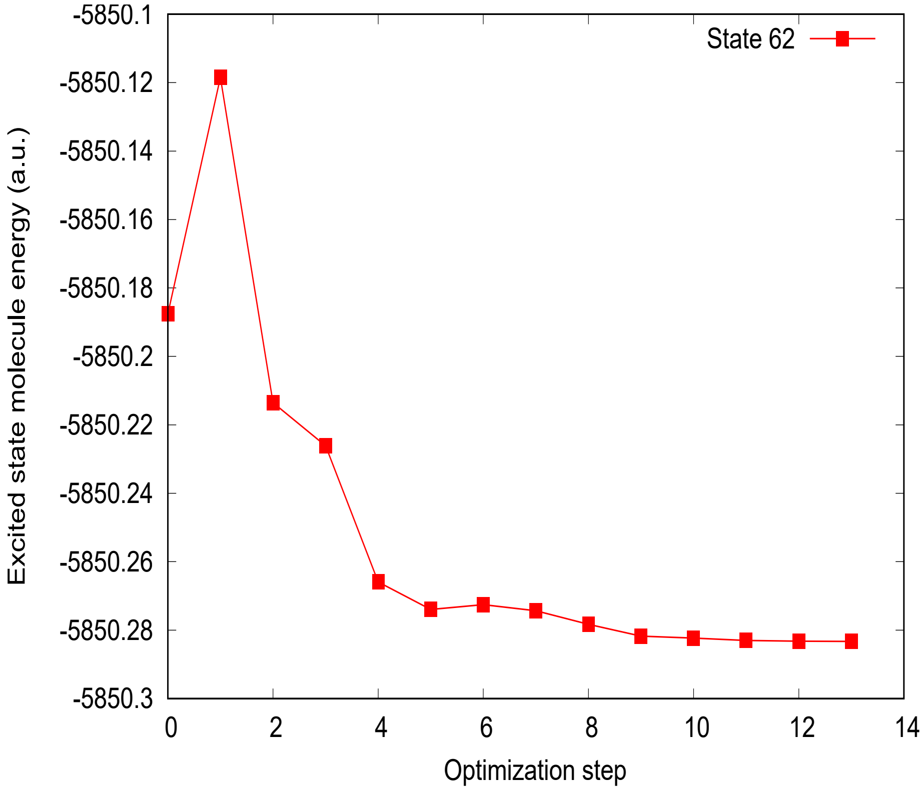


**Figure S9**: Excited state energy during optimization. Convergence values achieved at final step: Maximum force: 0.001105; RMS force: 0.000106 (converged); Maximum displacement:0.052343; RMS displacement: 0.010114.

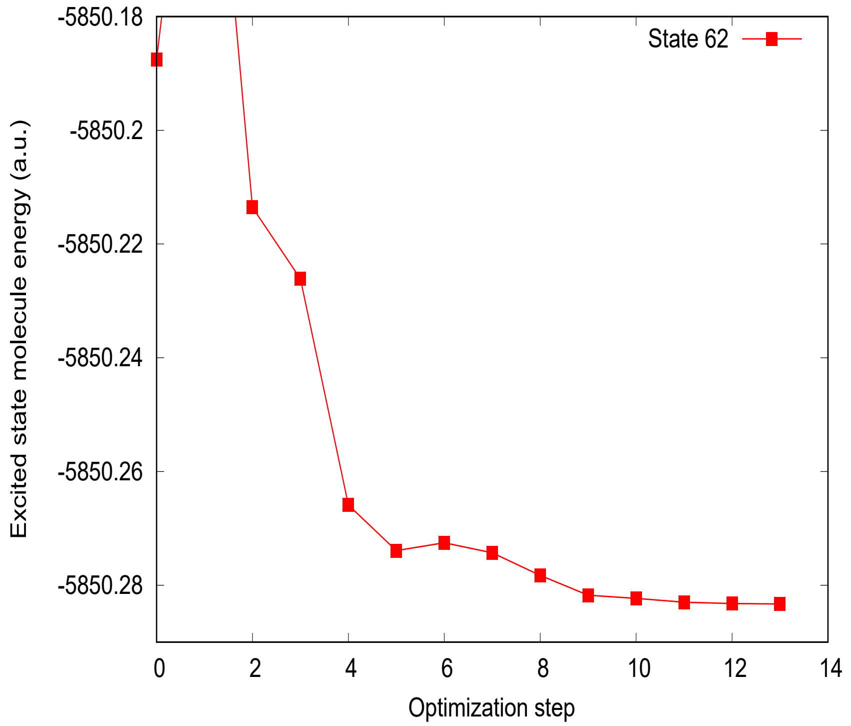


**Figure S10**: Excited state energy during optimization (expanded scale).

State 66:

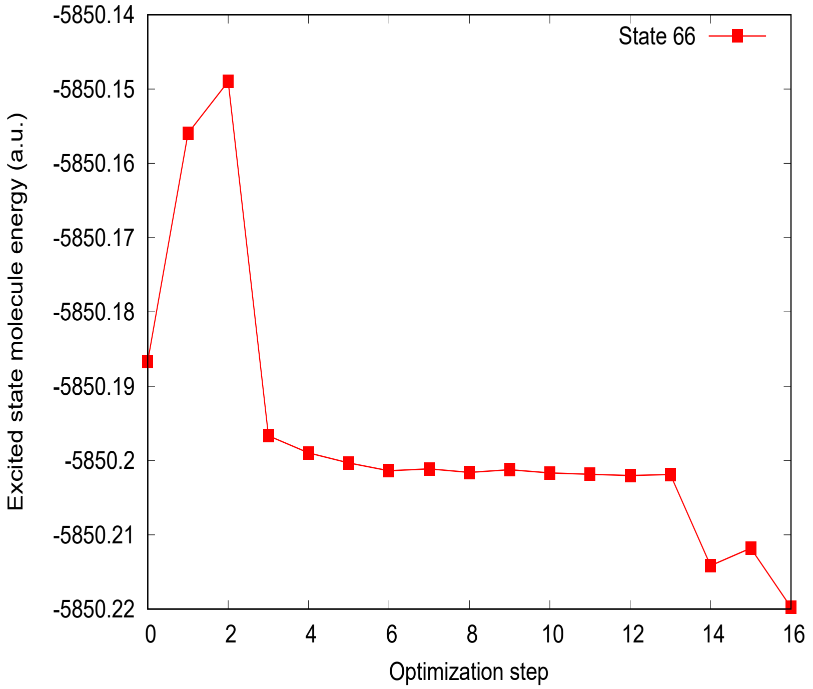


**Figure S11**: Excited state energy during optimization. Convergence values achieved at final step: Maximum force: 0.015359; RMS force: 0.000933; Maximum displacement: 1.443374; RMS displacement: 0.284491

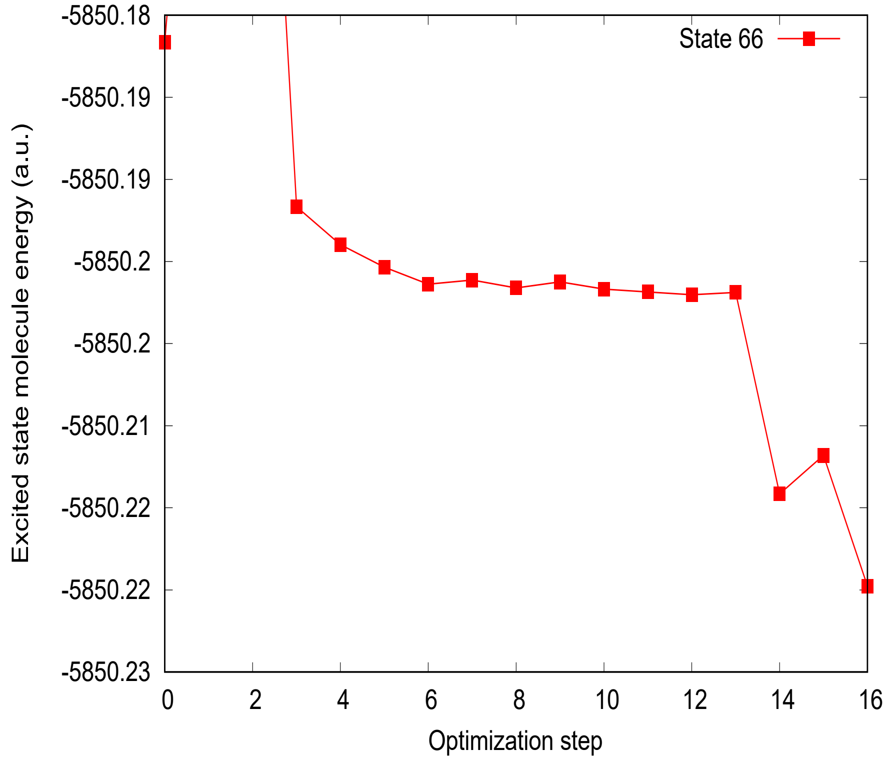


.

**Figure S12.** Excited state energy during optimization (expanded scale).

State 74:

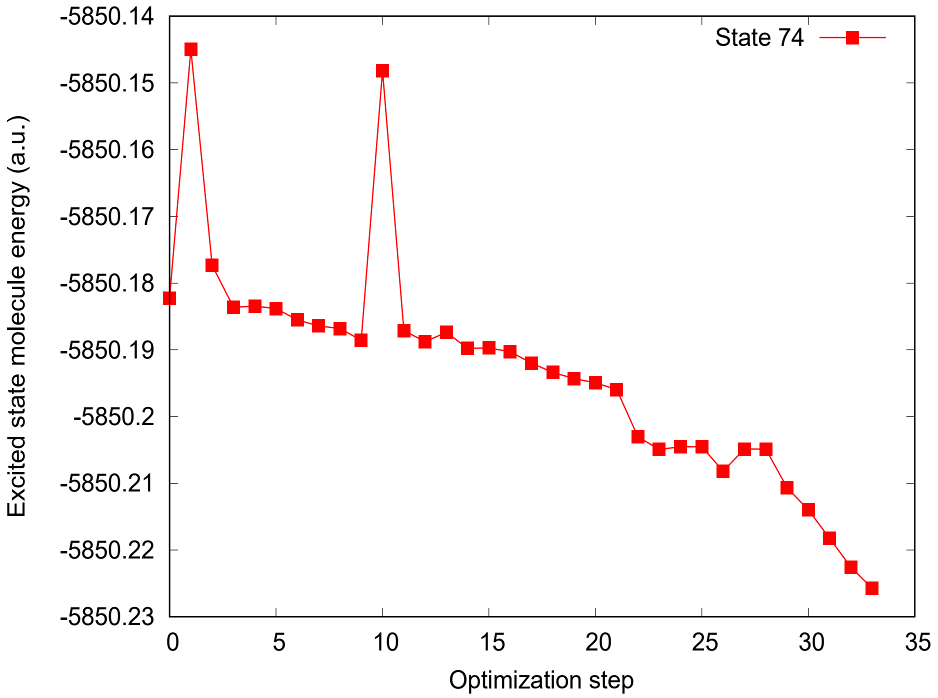


**Figure S13**: Excited state energy during optimization. Convergence values achieved at final step: Maximum force: 0.066001; RMS force: 0.003762; Maximum displacement: 1.037354; RMS displacement: 0.188578.

State 77:

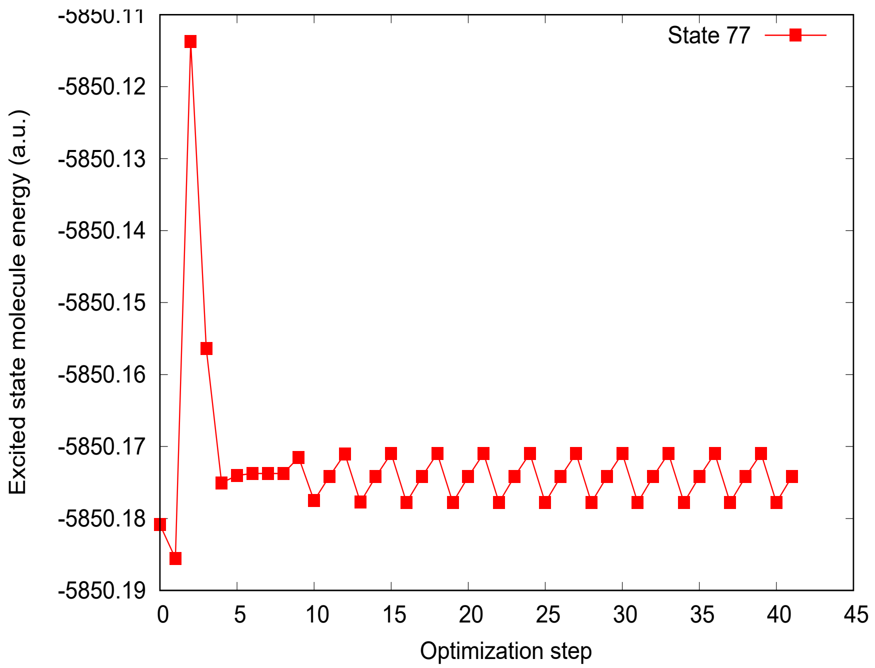


**Figure S14**: Excited state energy during optimization. Convergence values achieved at next to final (lowest energy) step: Maximum force: 0.025773; RMS force: 0.002540; Maximum displacement: 0.276725; RMS displacement: 0.042559. Note that the optimization ends up cycling among three energies. Examination of the geometries for these three energies shows the molecule shifting back and

forth between structures with small geometry differences but with no obvious differences (such as alignment of two rings).

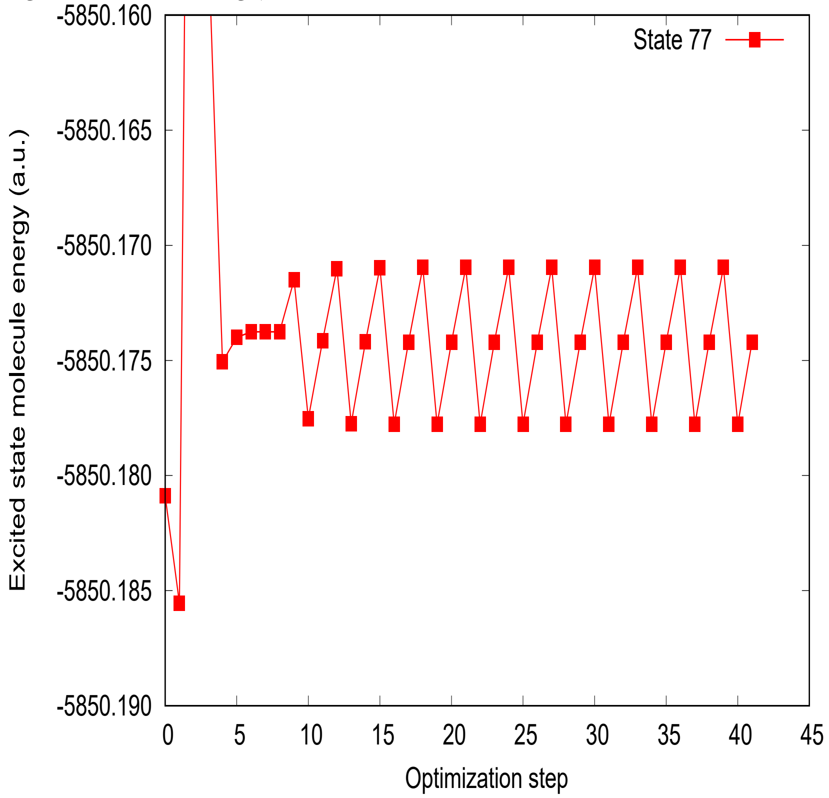


**Figure S15**: Excited state energy during optimization (expanded scale)

State 80:

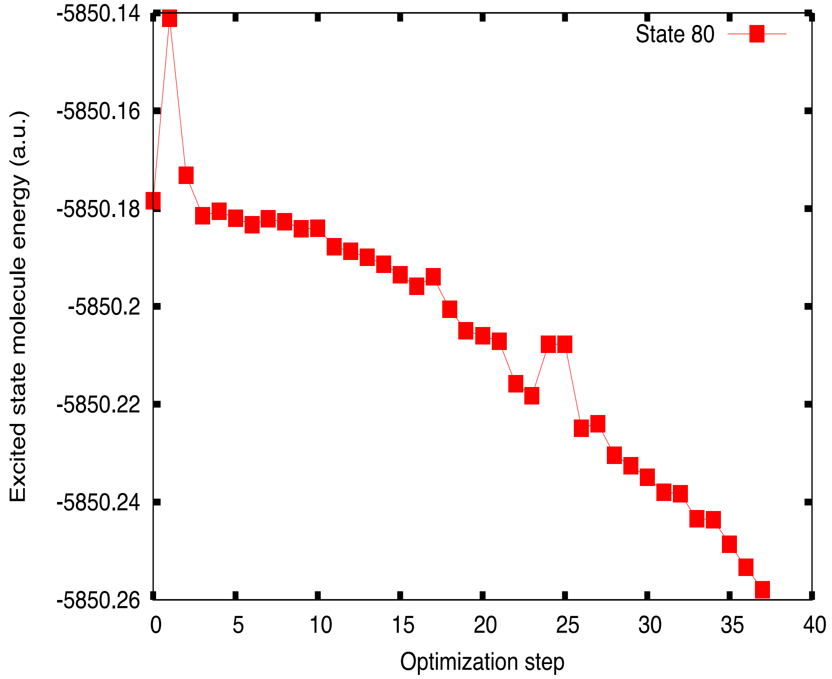


**Figure S16**: Excited state energy during optimization. Convergence values achieved at final step: Maximum force: 0.0.029008; RMS force: 0.003009; Maximum displacement: 1.347442; RMS displacement: 0.202959

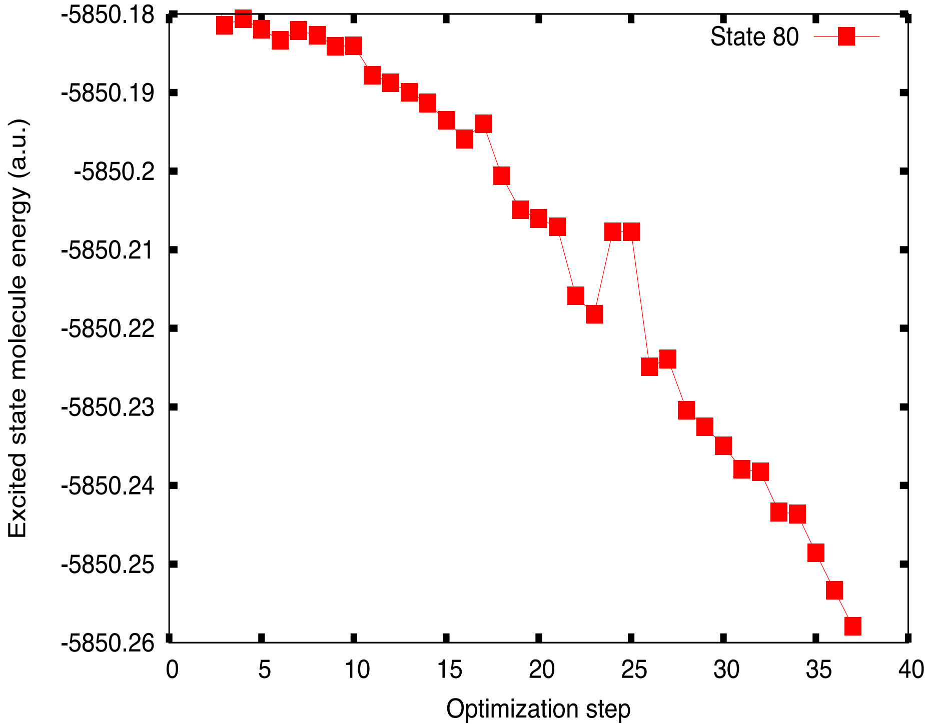


*Figure S17: Excited state energy during optimization (expanded scale).*

CF$_3$-styryl

State 57:

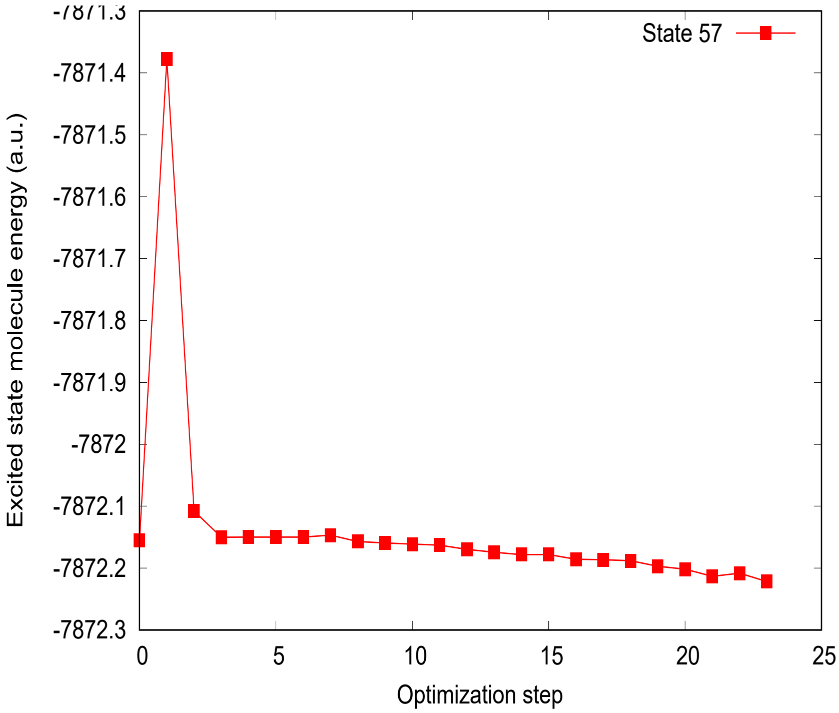


**Figure S18**: Excited state energy during optimization. Convergence values achieved at final step: Maximum force: 0.0.037363; RMS force: 0.003696; Maximum displacement: 1.420753; RMS displacement: 0.282626.

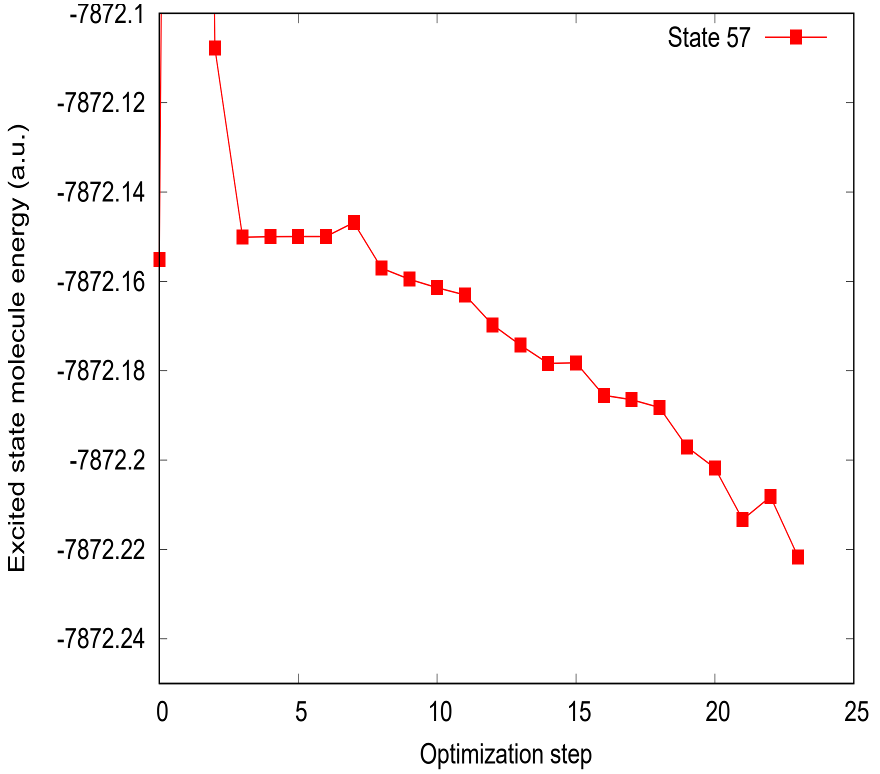


Figure S19: Excited state energy during optimization (expanded scale).

State 73:

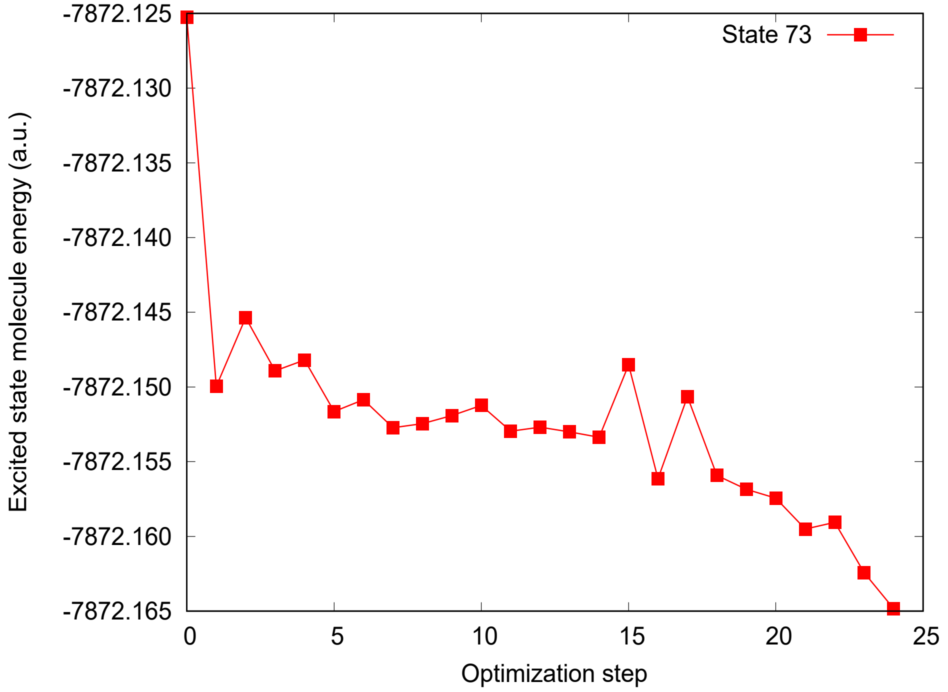


**Figure S20**: Excited state energy during optimization. Convergence values achieved at final step: Maximum force: 0.0.032637; RMS force: 0.004007; Maximum displacement: 0.513102; RMS displacement: 0.089769.

State 75:

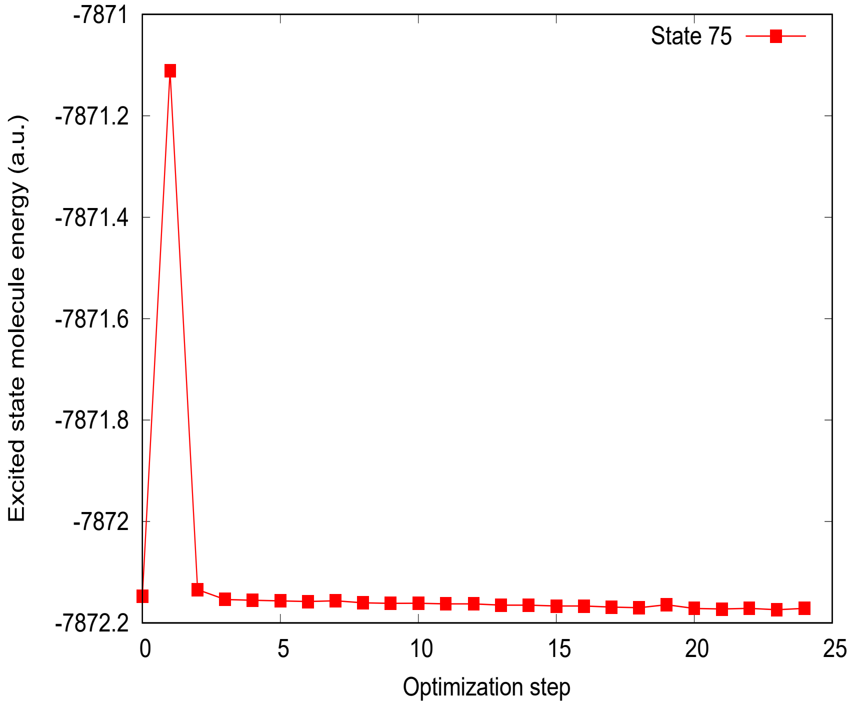


**Figure S21**: Excited state energy during optimization. Convergence values achieved at final step: Maximum force: 0.027480; RMS force: 0.002184; Maximum displacement: 0.895196; RMS displacement: 0.145767.

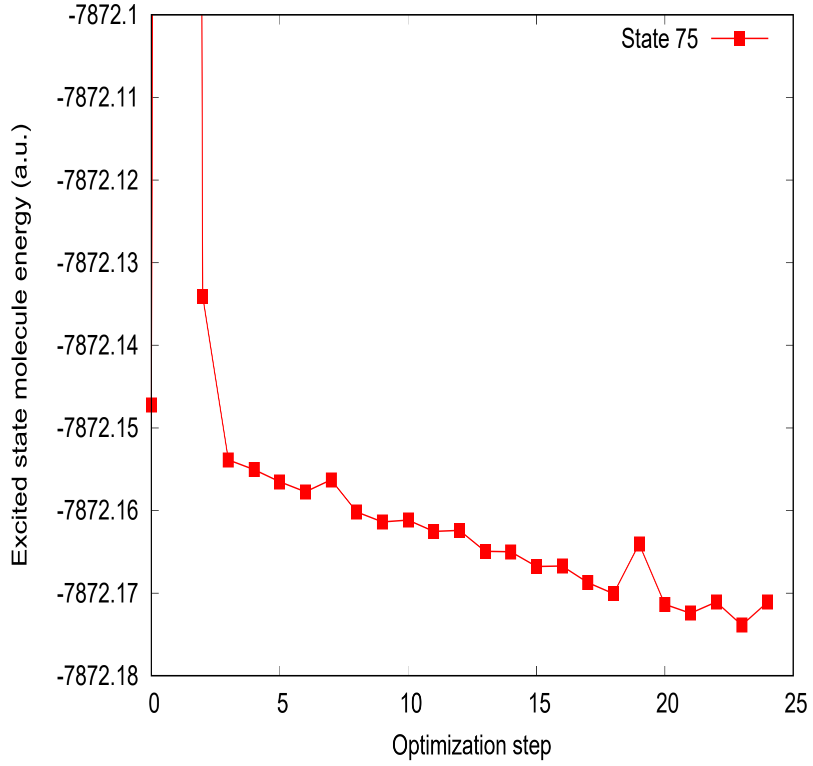


**Figure S22**: Excited state energy during optimization (expanded scale)

State 76:

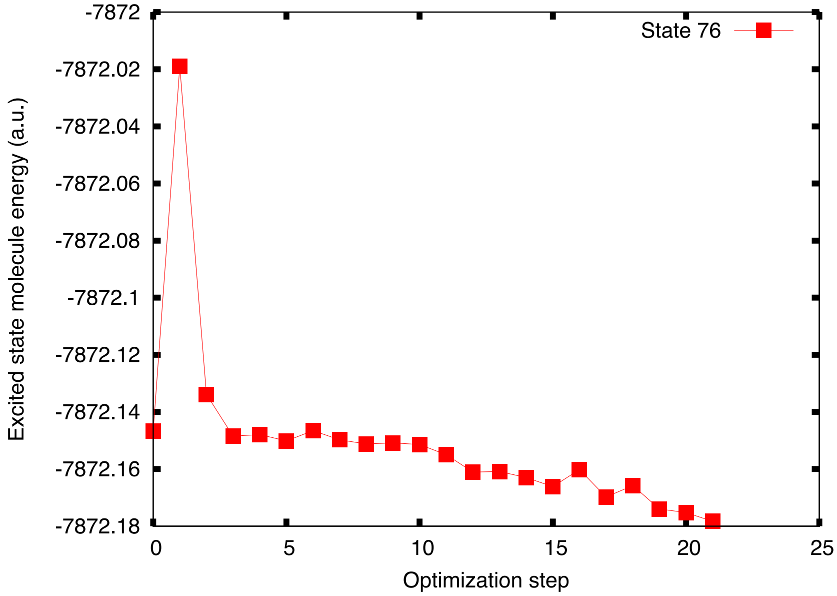


**Figure S23**: Excited state energy during optimization. Convergence values achieved at final step: Maximum force: 0.044068; RMS force: 0.003964; Maximum displacement: 0.482979; RMS displacement: 0.097400

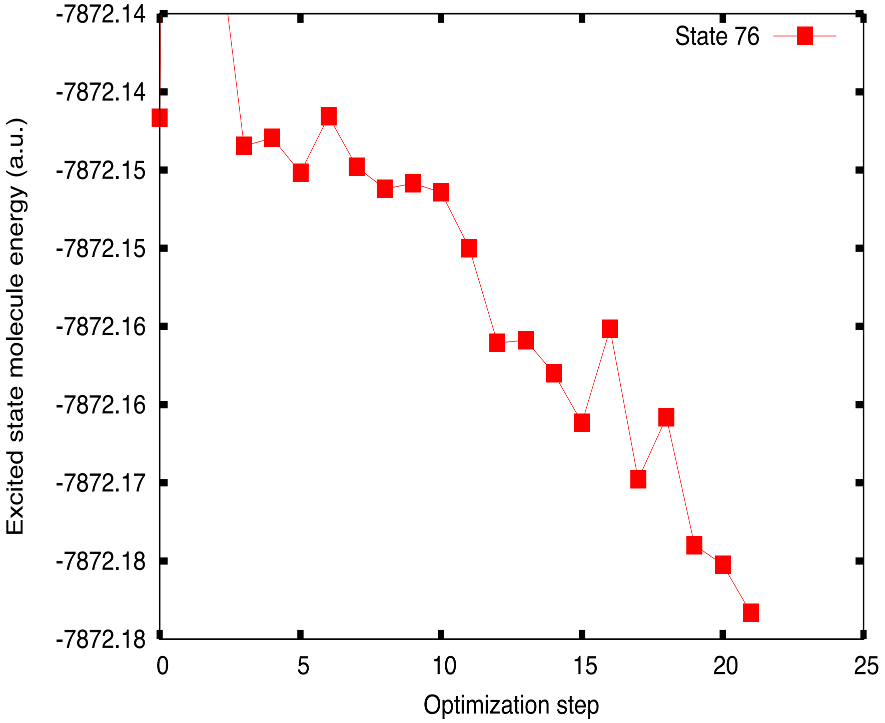


.

**Figure S24** -expanded: Excited state energy during optimization (expanded scale)

State 77:

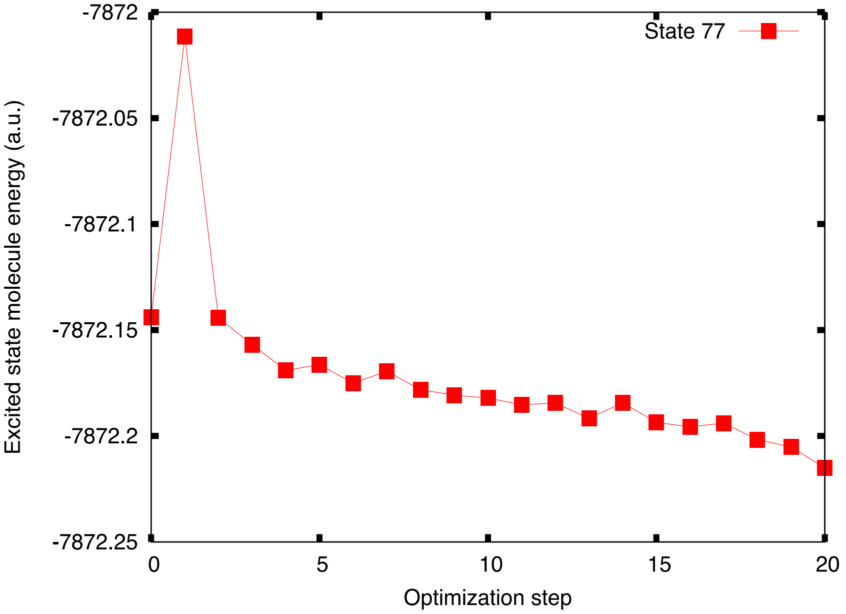


**Figure S25**: Excited state energy during optimization. Convergence values achieved at final step: Maximum force: 0.046488; RMS force: 0.003038; Maximum displacement: 2.025386; RMS displacement: 0.373846.

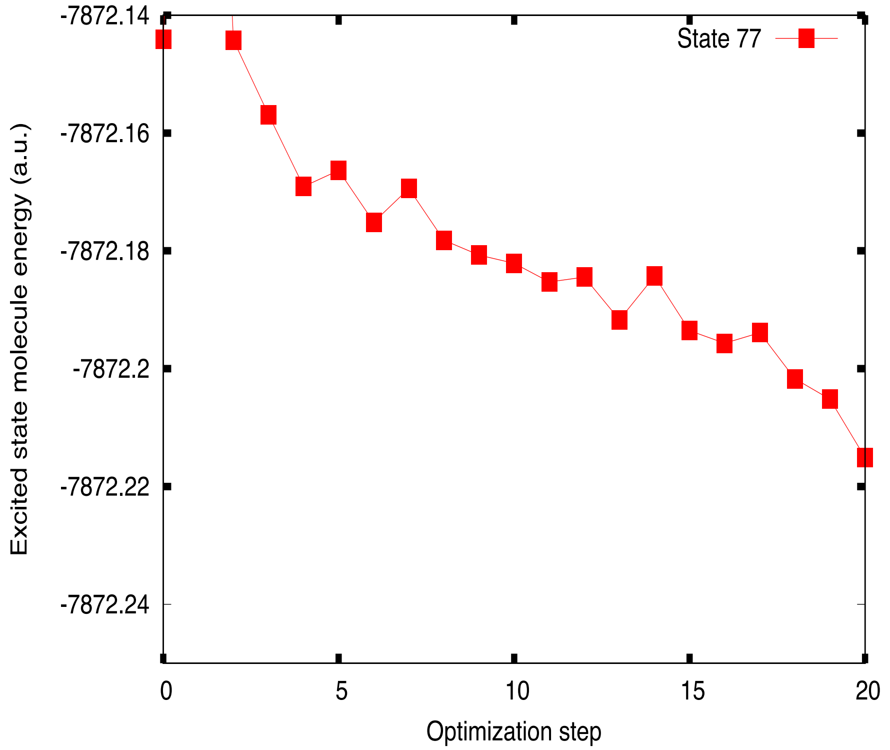


**Figure S26** -expanded: Excited state energy during optimization (expanded scale).
